# The Tris(pentafluorophenyl)methylium Cation: Isolation and Reactivity

**DOI:** 10.1002/anie.202203777

**Published:** 2022-05-16

**Authors:** Kurt F. Hoffmann, David Battke, Paul Golz, Susanne M. Rupf, Moritz Malischewski, Sebastian Riedel

**Affiliations:** ^1^ Fachbereich für Biologie, Chemie, Pharmazie Institut für Chemie und Biochemie—Anorganische Chemie Fabeckstraße 34/36 14195 Berlin Germany

**Keywords:** High Hydride Affinity, Perfluorinated Trityl Cation, Strong Oxidizer, Superacids, Weakly Coordinating Anions

## Abstract

Herein, we present two different routes for the synthesis of the perfluorinated trityl cation, which allowed the handling of the free, uncoordinated species in organic solvents for the first time. The usage of the weakly coordinating anion [Al(OTeF_5_)_4_]^−^ and its derivatives allows the characterization of this species by NMR spectroscopy and most importantly by single‐crystal X‐ray diffraction. The high hydride ion affinity of the cation is shown by hydrogen abstraction from isobutane. Furthermore, cyclic voltammetry reveals its oxidative potential which is supported by the reaction with tris(4‐bromophenyl)amine, giving rise to the formation of the ammoniumyl radical cation, also known as “magic blue”.

## Graphical abstract

Two brave knights worrying for their hydrides and electrons are caught in the storm of a small tritylium and a giant perfluorotritylium windmill. Herein, the structural proof of the perfluorinated trityl cation and its remarkable hydride ion affinity and oxidation potential compared to its non‐fluorinated analogue are reported.

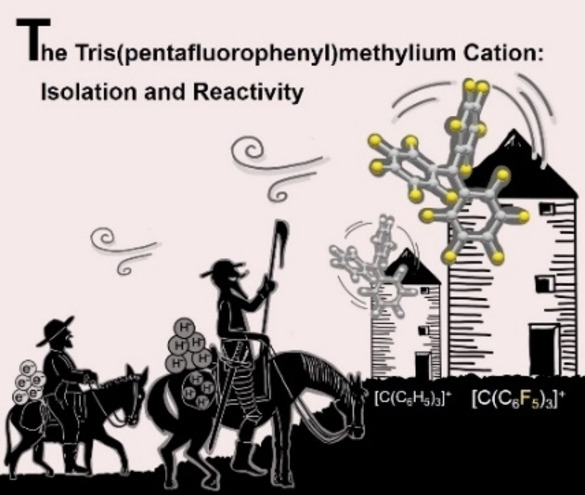

The trityl cation [C(C_6_H_5_)_3_]^+^ is known as a versatile compound for hydride and methide abstraction reactions and in combination with weakly coordinating anions it finds widespread application in modern chemistry, e.g. as co‐catalyst in olefin polymerization^[1]^ or as key reagent in the generation of silylium cations.^[2]^ More recently, the application of tritylium cations as components in frustrated radical pairs was reported.^[3]^ Still, they are viable reagents for hydride abstraction and recently enabled the isolation of a phosphorus dication.^[4]^ Its reactivity can be even further enhanced by replacing the H atoms of the phenyl groups by F atoms.

A theoretical study by Dutton et al.^[5]^ on the Lewis acidity of the perfluorinated trityl cation in terms of calculated ion affinities towards small Lewis bases showed a strong increase of the Lewis acidity compared to its non‐fluorinated analogue and even surpassed the isoelectronic tris(pentafluorophenyl) borane B(C_6_F_5_)_3_. This becomes evident when comparing the calculated gas‐phase fluoride ion affinity (FIA) and hydride ion affinity (HIA) of the perfluorinated trityl cation (FIA: 697 kJ mol^−1^; HIA: 955 kJ mol^−1^) with its non‐fluorinated analogue (FIA: 599 kJ mol^−1^; HIA: 801 kJ mol^−1^) and B(C_6_F_5_)_3_ (FIA: 403 kJ mol^−1^; HIA: 455 kJ mol^−1^). The effect of fluorination of the phenyl groups was further studied by Stephan et al. as well as Mayr and Horn. They reported on an increase of the global electrophilicity index^[6]^ and the rate of hydride transfer reactions,^[7]^ respectively.

The first formation and NMR spectroscopic investigations of the perfluorinated trityl cation were reported independently by the groups of Filler and Olah in the 1960s.[^8^, ^9^] Their synthesis was based on the reaction of the alcohol C(C_6_F_5_)_3_OH with neat oleum or “magic acid” (HSO_3_F/SbF_5_). Nevertheless, it was not possible to isolate a salt of this compound.

Dutton et al. recently followed up on this approach using triflic acid.^[10]^ While they observed the successful formation of the perfluorinated trityl cation [C(C_6_F_5_)_3_]^+^ in neat triflic acid, only the contact ion pair C(C_6_F_5_)_3_OTf is formed in organic solvents such as *ortho*‐dichlorobenzene. Still, this compound possessed a certain reactivity as a hydride acceptor which they proved by the reaction of C(C_6_F_5_)_3_OTf with triethylsilane resulting in the partial formation of tris(pentafluorophenyl)methane HC(C_6_F_5_)_3_.

In contrast to the previous described routes for the synthesis of the perfluorinated trityl cation, our approach starts from tris(pentafluorophenyl)methyl chloride **1** and the Brønsted superacid [H‐C_6_F_2_H_4_][Al(OTeF_5_)_4_] (cf. Figure 1). This Brønsted superacid is formed by reacting AlEt_3_ and HOTeF_5_ in *ortho‐*difluorobenzene (*o*‐DFB) and is generally used to generate cations in two ways; via the protonation of weak bases and via the elimination of gaseous HCl out of chloride sources.[^11^, ^12^, ^13^] Taking advantage of this reactivity, we added chloride **1** to a solution of [H‐C_6_F_2_H_4_][Al(OTeF_5_)_4_] followed by an immediate color change from yellow to an intense purple as well as the evolution of HCl gas (cf. Scheme 1). At the same time, the weakly coordinating properties of the anion [Al(OTeF_5_)_4_]^−^ stabilizes the perfluorinated trityl cation.

**Figure 1:**
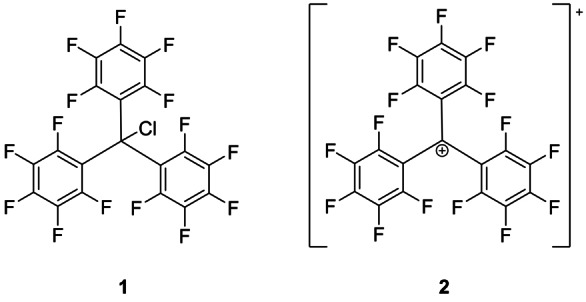
Lewis structures of the precursor tris(pentafluorophenyl)methyl chloride **1** and the product tris(pentafluorophenyl)methylium **2**.

**Scheme 1:**
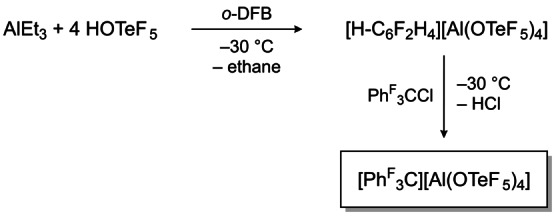
Synthesis of the perfluorinated trityl cation via a Brønsted acidic route.

Analysis of the solution by low‐temperature NMR spectroscopy confirmed the successful formation of the desired species. In the ^19^F NMR spectrum the signals observed at a chemical shift of −112.6, −127.7 and −154.3 ppm with relative integrals of 3 : 6 : 6 correspond to the *para‐*, *ortho‐* and *meta‐*fluorine atoms of the perfluorinated trityl cation **2** and are in agreement with already reported spectra of the cation in “magic acid” and neat triflic acid.[^9^, ^10^] Furthermore, a signal set corresponding to an AB_4_ pattern appears at chemical shifts of −40.1 and −47.2 ppm with a ^2^ *J*_FF_ coupling constant of 186 Hz belonging to the pentafluoro‐orthotellurate groups of the anion [Al(OTeF_5_)_4_]^−^.[^11^, ^12^] In the ^27^Al NMR spectrum the signal of the anion is found at 47 ppm.[^11^, ^12^] Repeating the NMR experiments at room temperature leads to the following changes: In the ^19^F NMR spectrum the signals of cation **2** are broadened and a new set of signals at −142.9, −154.3 and −162.7 ppm appear. This is assigned to the already reported decomposition product HCPh^F^_3_.^[10]^ Its formation probably occurs via hydrogen abstraction of the cation from the solvent *o*‐DFB. After 24 hours, the signals of the cation are not observable anymore. Also, this decomposition reaction slowly proceeds at −40 °C, since needle‐shaped single crystals of HCPh^F^_3_ grew in a concentrated solution of [C(C_6_F_5_)_3_][Al(OTeF_5_)_4_] in *o*‐DFB (cf. Figure 2).^[14]^ Attempts to prepare the cation by reacting perfluorotrityl chloride **1** and pentafluoro‐orthotelluric acid did not show any reaction. This underlines that a rather strong Brønsted superacid is required to achieve the HCl elimination in this reaction.

**Figure 2:**
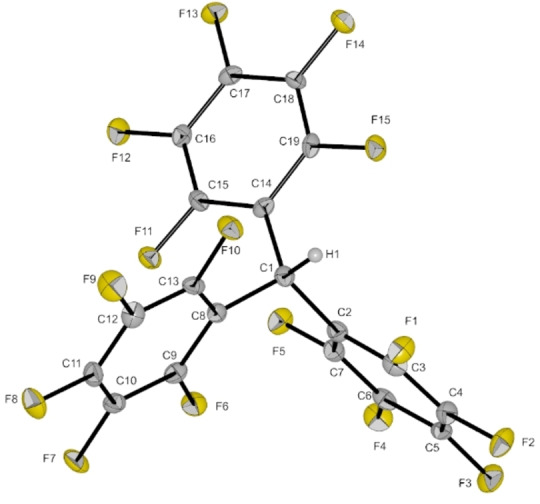
Molecular structure of HC(C_6_F_5_)_3_ in the solid state. Thermal ellipsoids set at 50 % probability. Selected bond lengths [pm] and angles [°]: C1–C2 153.5(4), C1–C8 152.7(4), C1–C14 153.6(4); C2‐C1‐C14 113.5(2), C8‐C1‐C2 114.6(2), C8‐C1‐C14 113.4(2).

Nevertheless, by layering a solution of [C(C_6_F_5_)_3_][Al(OTeF_5_)_4_] in a mixture of *o*‐DFB and dichloromethane with *n*‐pentane at −80 °C it was possible to obtain dark purple, plate‐shaped single crystals suitable for x‐ray diffraction. The compound crystallizes in the monoclinic space group *C*2/*c* (cf. Figure 3).^[14]^ Similar to the non‐fluorinated trityl cation, the phenyl rings are arranged in a propeller‐like manner. The sum of angles around the central carbon atom (C1) is 359.9(9)°, which is in line with free trityl cations.^[15]^ The closest cation‐anion contact lies between a *para*‐fluorine atom of a phenyl ring and a fluorine atom of a pentafluoro‐orthotellurate group (*d*(F3−F15)=268.7(10) pm) and is slightly shorter than the sum of the van‐der‐Waals radii (*d*(F−F)_vdW_=294 pm). The distance between C1 to the closest anionic fluorine atom (F10) is 289.7(3) pm. This distance is slightly decreased compared to the non‐fluorinated trityl analogue [C(C_6_H_5_)_3_][Al(OTeF_5_)_4_] (closest *d*(C⋅⋅⋅F): 303.3(3) pm) and is probably caused by the enhanced electrophilicity of cation **2**.^[11]^ The average dihedral angles of the phenyl rings in cation **2** are with 36.78° slightly larger compared to the non‐fluorinated analogue with an average dihedral angle of 31.14°.

**Figure 3:**
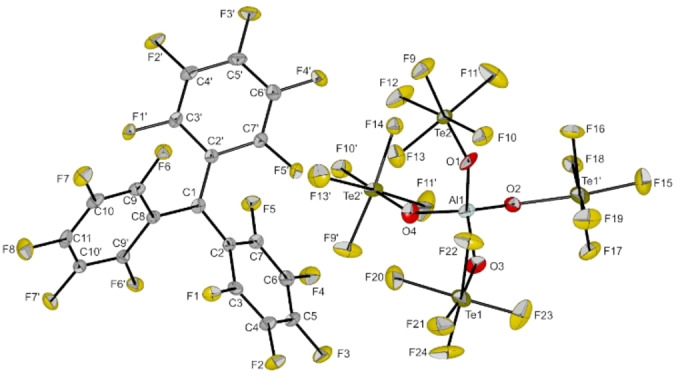
Molecular structure of [C(C_6_F_5_)_3_][Al(OTeF_5_)_4_] in the solid state. Thermal ellipsoids set at 50 % probability. Selected bond lengths [pm] and angles [°]: C1–C2 143.2(6), C1–C8 144.9(10); C2‐C1‐C8 119.8(3), C2‐C1‐C2′ 120.5(6), C2′‐C1‐C8 119.8(3).

In a second approach the Lewis superacid Al(OTeF_5_)_3_ was used as starting material instead of the Brønsted superacid [H‐C_6_F_2_H_4_][Al(OTeF_5_)_4_]. By dissolving solid [Al(OTeF_5_)_3_]_2_ in an excess of SO_2_ClF a clear solution is formed. Upon addition of solid perfluorotrityl chloride **1**, an immediate change of color to intense purple is observed (cf. Scheme 2).

**Scheme 2:**
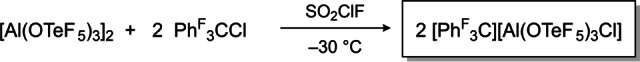
Synthesis of the perfluorinated trityl cation via a Lewis acidic route.

An analysis of the reaction mixture by low‐temperature NMR spectroscopy showed the successful formation of the perfluorinated trityl cation. The formed anion [Al(OTeF_5_)_3_Cl]^−^ is presumably in an equilibrium between the fully pentafluoro‐orthotellurate‐substituted anion [Al(OTeF_5_)_4_]^−^ and an anion with two chlorido and two pentafluoro‐orthotellurate ligands [Al(OTeF_5_)_2_Cl_2_]^−^. The ^19^F NMR spectrum shows again the signals of the cation **2** at −109.6, −125.5 and −152.3 ppm. Additionally, three different AB_4_ signal sets are observed between −35 and −50 ppm. They correspond to the equilibrating anions [Al(OTeF_5_)_4−*x*_Cl_*x*_]^−^ (*x*=0,1,2). The ^27^Al NMR also shows three different signals, assigned to [Al(OTeF_5_)_4_]^−^ at 48 ppm, [Al(OTeF_5_)_3_Cl]^−^ at 65 ppm and [Al(OTeF_5_)_2_Cl_2_]^−^ at 80 ppm.^[11]^ The ^13^C NMR spectrum shows the signals of cation **2**. Surprisingly, the signal of the central carbon atom at 175 ppm is 36 ppm upfield‐shifted compared to the non‐fluorinated trityl cation.^[11]^ This phenomenon may occur due to π donation of the adjacent fluorine atoms to the central carbon atom and are in agreement with findings of Dutton et al.^[10]^ Warming the reaction mixture to room‐temperature did not lead to a decolorization. Still, the corresponding NMR spectra at room temperature indicate decomposition of the compound within 15 minutes. The successful formation of the cation via a Lewis acidic reaction pathway is in contrast to previous experimental findings, in which only an reaction at the *para*‐fluorine atom instead of an attack at the chloride bound to the central carbon atom was observed when halide abstraction reagents were used.^[10]^ Attempts to isolate the compound by evaporation of all volatiles led to a viscous, dark purple oil, which could not be further characterized so far.

Quantum‐chemical calculations were performed to understand the electronic properties of the perfluorinated trityl cation. In the top part of Figure 4, the plotted electrostatic potential of the perfluorinated and non‐fluorinated trityl cation is shown. In the case of the [C(C_6_H_5_)_3_]^+^ ion the positive electrostatic potential is located at the central carbon atom as well as on the peripheral hydrogen atoms, while the negative potential is located near the aromatic ring. In the case of the [C(C_6_F_5_)_3_]^+^ ion the distribution of the electrostatic potential is changed: All carbon atoms possess a rather positive electrostatic potential. Due to the strong electron‐withdrawing properties of the fluorine substituents, the peripheral F atoms accumulate a more negative electrostatic potential. A closer look on the fluorine substituents reveals the lowest electrostatic potential at the *meta‐*fluorine atoms compared to the *para*‐ and *ortho*‐fluorine atoms. The bottom part of Figure 4 depicts the lowest unoccupied molecular orbitals (LUMO) of [C(C_6_H_5_)_3_]^+^ and [C(C_6_F_5_)_3_]^+^, mainly centered on the unoccupied p‐orbital of the central carbon atom. The LUMO energy of [C(C_6_F_5_)_3_]^+^ is lowered by 1.37 eV compared to [C(C_6_H_5_)_3_]^+^, which reflects the increased electrophilicity. Furthermore, the adiabatic ionization energy of the neutral radical species C(C_6_F_5_)_3_⋅ and C(C_6_H_5_)_3_⋅ show a difference of 1.45 eV. Consequently, this should lead to an increased oxidation potential of the fluorinated trityl cation compared to the non‐fluorinated analogue.

**Figure 4:**
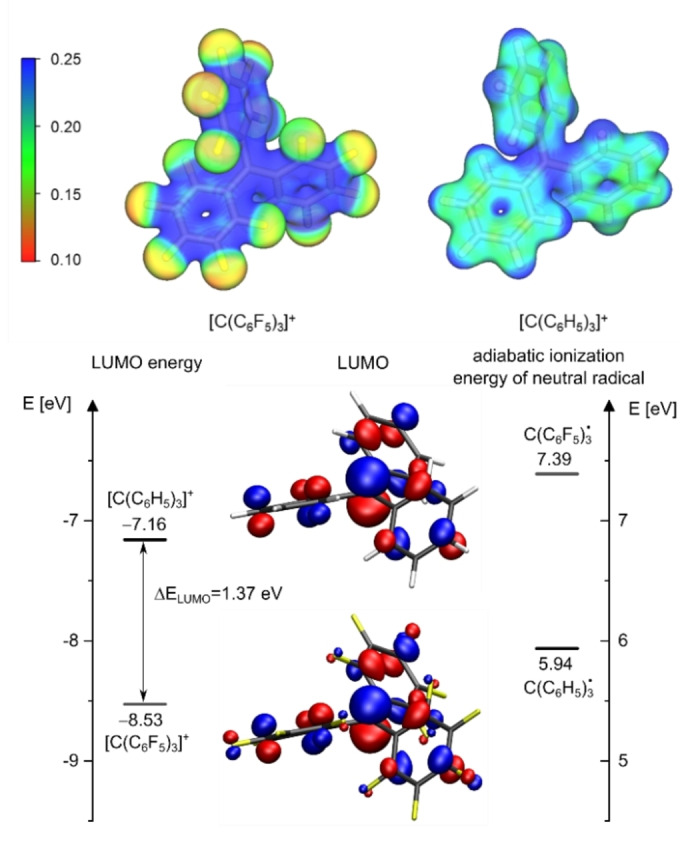
Top: Plotted electrostatic potential of perfluorinated and non‐fluorinated trityl cations (in *E*_H_, isovalue: 0.025). Bottom: Plotted Lowest Unoccupied Molecular Orbitals of [C(C_6_F_5_)_3_]^+^ and [C(C_6_H_5_)_3_]^+^ along with the calculated difference of LUMO energy and the adiabatic ionization energy. Calculations were performed at RI‐B3LYP/def2‐TZVPP level of theory.

As quantum‐chemical calculations of Couchman et al. already indicated, a high hydride ion affinity is expected for the perfluorinated trityl cation (calculated gas‐phase hydride affinities: [C(C_6_F_5_)_3_]^+^=955 kJ mol^−1^ vs. [C(C_6_H_5_)_3_]^+^=801 kJ mol^−1^).^[5]^ This is experimentally supported by the observed formation of the side‐product tris(pentafluorophenyl)methane in the crystallization attempts of compound [C(C_6_F_5_)_3_][Al(OTeF_5_)_4_]. In order to elaborate on the experimental hydride affinity of cation **2**, the generation of the *tert*‐butyl cation by means of a hydrogen abstraction on isobutane was undertaken. A similar reaction was shown by Reed and co‐workers, who prepared a *tert*‐butyl cation by treating the very strong methylation reagent Me(CHB_11_Me_5_Br_6_) with isobutane and thereby formed methane and the *tert*‐butyl cation.^[16]^ Therefore, a solution of [C(C_6_F_5_)_3_][Al(OTeF_5_)_3_Cl] in SO_2_ClF was prepared by the prior described reaction (cf. Scheme 2) and treated with an excess of isobutane. The reaction was then monitored by low‐temperature NMR spectroscopy (cf. Scheme 3). The recorded spectra can be found in the Supporting Information.

**Scheme 3:**
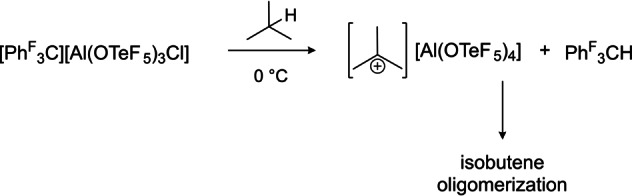
Reaction of the perfluorinated trityl cation with isobutane performed in SO_2_ClF.

While at −60 °C no reaction occurred, warming the reaction mixture to 0 °C gave insight into the reaction progress. In the ^19^F NMR spectrum the decrease of the signals corresponding to cation **2** is observed while a new set of signals at −142.9, −154.9 and −163.0 ppm emerges, which belongs to the formation of HCPh^F^_3_. The singlet of HCPh^F^_3_ in the ^1^H NMR spectrum appears at 6.8 ppm. In the ^27^Al NMR spectrum the signals of the mixed anions [Al(OTeF_5_)_3_Cl]^−^ and [Al(OTeF_5_)_2_Cl_2_]^−^ vanished, while the homoleptic anion [Al(OTeF_5_)_4_]^−^ remains stable. The signals of the isobutane at 2.2 and 1.5 ppm in the ^1^H NMR spectrum as well as at 24.3 and 23.6 ppm in the ^13^C NMR spectrum are severely broadened, indicating a dynamic process. These observations confirm a successful hydride abstraction by the [C(C_6_F_5_)_3_]^+^ ion while the formed *tert*‐butyl cation might undergo a dynamic exchange with the remaining isobutane or even the present chloride anions of the mixed anion species. Such processes already have been described in literature.^[17]^ Nevertheless, these experiments clearly underline the high hydride ion affinity of cation **2** by cleanly converting the cation to the perfluorinated trityl methane. For further details see Supporting Information.

The influence of the fluorine substitution pattern of the phenyl rings in trityl cations on its electrophilicity and stability has been already discussed in literature.^[18]^ However, there are no reports on the change of its oxidative potential by introduction of more electron‐withdrawing substituents. The tris(pentafluorophenyl)‐methyl radical has already been mentioned in a previous publication and was synthesized by the reduction of the [C(C_6_F_5_)_3_]^+^ cation with TiCl_3_.^[19]^ Therefore, it should be feasible to analyze a solution of [C(C_6_F_5_)_3_][Al(OTeF_5_)_4_] in *o*‐DFB by cyclic voltammetry. The experiment gave an irreversible oxidation wave at *E*_pa_=1.11 V against Fc/Fc^+^ (cf. Figure 5), which surpasses the formal oxidative potential of its non‐fluorinated analogue by a remarkable 1.22 V (cf. Table 1). This strong increase of the oxidative potential can be explained by the increase of the ionization energy, induced by the fluorine substitution (see above). A similar effect on the oxidation potential was observed when the group of Krossing et al. investigated fluorine‐substituted dihydrophenanzine derivatives.^[20]^

**Figure 5:**
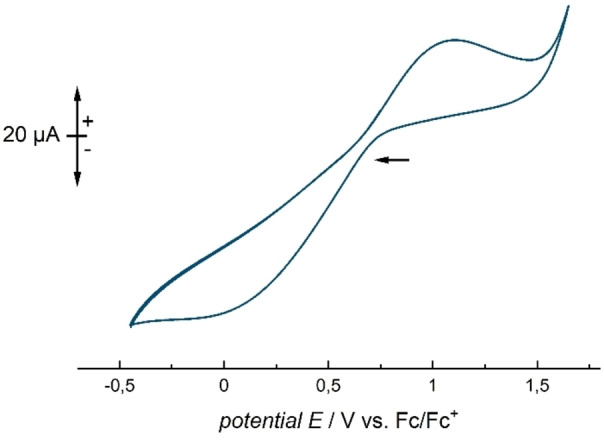
Cyclic voltammogram of a 0.078 M solution of [C(C_6_F_5_)_3_][Al(OTeF_5_)_4_] in *ortho*‐difluorobenzene at −35 °C. Scan rate: 100 mV s^−1^. Irreversible oxidation wave at 1.11 V.

**Table 1:** Formal redox potentials (V vs. Fc/Fc^+^) of selected compounds.

Oxidant	Solvent	*E*°^[a]^
[N(C_6_H_4_Br‐2,4,6)_3_]^+^	MeCN	1.40^[21]^
[N(C_6_H_4_Br‐2,4)_3_]^+^	MeCN	1.18^[21]^
[CPh_3_^F^]^+^	*o‐*DFB	1.11
[NO]^+^	CH_2_Cl_2_	1.00^[22]^
[N(C_6_H_4_Br‐4)_3_]^+^	CH_2_Cl_2_	0.74^[21]^
[FeCp_2_]^+^		0.00
[CPh_3_]^+^	MeCN	−0.11^[23]^

[a] Values have been corrected accordingly: Fc/Fc^+^ *E*_1/2_=0.31 V vs. SCE (0.56 V vs. NHE).

In order to show the oxidative potential of the cation **2** experimentally, it was reacted with ferrocene and tris(4‐bromophenyl)amine, respectively. In the case of ferrocene addition, an immediate color change to green is observed, due to the formation of the ferrocenium cation [FeCp_2_]^+^. The addition of tris(4‐bromophenyl)amine to a solution of [C(C_6_F_5_)_3_][Al(OTeF_5_)_4_] in *o*‐DFB lead to a deep‐blue colored reaction mixture, indicating the successful formation of the ammoniumyl radical cation (cf. Scheme 4), also known as “magic blue”. Subsequently the tris(pentafluorophenyl)methyl radical Ph^F^_**3**_C⋅ must have been formed. For further characterization, both reaction mixtures were analyzed by EPR spectroscopy. The EPR spectrum of the reaction of ferrocene with [C(C_6_F_5_)_3_][Al(OTeF_5_)_4_] gave one broadened signal with a *g* value of 2.0031, which corresponds to the Ph^F^_3_C⋅ radical and is in line with the literature reported *g* value for this species.^[19]^ The signal of the [FeCp_2_]^+^ cation is not expected, since the measurement was performed at room temperature and it is known that this species can only be observed at temperatures below 78 K.^[24]^ The EPR spectrum of the reaction between [C(C_6_F_5_)_3_][Al(OTeF_5_)_4_] and the tris(4‐bromophenyl)amine shows two overlapping broad signals, which correspond to the ammoniumyl radical cation and the perfluorinated trityl radical (see Figures S16). This is in agreement with literature, where a severely broadened signal for the tris(4‐bromophenyl)ammoniumyl radical cation was reported.^[25]^

**Scheme 4:**
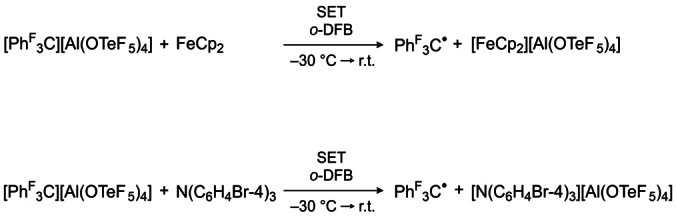
Reaction of the perfluorinated trityl cation with ferrocene (top) and tris(4‐bromophenyl)amine (bottom).

In conclusion, we report on two novel synthesis routes for the preparation of the perfluorinated trityl cation [C(C_6_F_5_)_3_]^+^. By using the weakly coordinating anion [Al(OTeF_5_)_4_]^−^ it was now possible to structurally characterize this interesting species. In conjunction with theoretical and experimental methods like EPR, NMR and CV the cation [C(C_6_F_5_)_3_]^+^ is further investigated. These new synthetic routes allow it to handle this delicate species in its free form in organic solvents such as *o*‐DFB. Moreover, we have experimentally shown its remarkable hydride ion affinity and its high oxidation potential.

## Note Added in Proof

Since the publication of this work as an Accepted Article, an article by Ozerov et al. has been published, which reports on the synthesis and reactivity of partially fluorinated trityl cation salts.^[25]^

## Conflict of interest

The authors declare no conflict of interest.

## Supporting information

As a service to our authors and readers, this journal provides supporting information supplied by the authors. Such materials are peer reviewed and may be re‐organized for online delivery, but are not copy‐edited or typeset. Technical support issues arising from supporting information (other than missing files) should be addressed to the authors.

Supporting Information

Supporting Information

Supporting Information

## Data Availability

The data that support the findings of this study are available from the corresponding author upon reasonable request.
