# Supplementary material for: The Tris(pentafluorophenyl)methylium Cation: Isolation and Reactivity
Source: Angew Chem Int Ed Engl. 2022 May 16;61(28):e202203777. doi: 10.1002/anie.202203777 (PMC9401592; doi:10.1002/anie.202203777)
Supplement: Supplementary file 3 — Supporting Information [file ANIE-61-0-s001.pdf]

Angewandte Chemie
Eine Zeitschrift der Gesellschaft Deutscher Chemiker

Supporting Information

The Tris(pentafluorophenyl)methylium Cation: Isolation and Reactivity

*K. F. Hoffmann, D. Battke, P. Golz, S. M. Rupf, M. Malischewski, S. Riedel**

Table of Contents

Experimental Procedures 2
NMR Spectra 4
Variable-Temperature NMR Spectra 10
EPR Spectra 13
Crystal data 14
Summary of crystal data and refinement results 14
$C(C_6F_5)_3H$ 15
$[C(C_6F_5)_3][Al(OTeF_5)_4]$ 17
Quantum-chemical calculations 20
CPh_3 radical 20
$[CPh_3]^+$ cation 21
CPh^F_3 radical 22
$[CPh^F_3]^+$ cation 23
$HCPh^F_3$ 25
References 26
Author Contributions .... **Fehler! Textmarke nicht definiert.**

Experimental Procedures

All preparative work was carried out using standard Schlenk techniques. Glassware was greased with Triboflon III. The pentafluoroorthotelluric acid $HOTeF_5$[1] and $[Al(OTeF_5)_3]_2$[2] were prepared as described elsewhere. All solid materials were handled inside a glove box with an atmosphere of dry argon (O_2 < 0.5 ppm, H_2O < 0.5 ppm). All solvents were freshly dried with CaH_2 before use and stored on molecular sieve. NMR spectra were recorded on a JEOL 400 MHz ECS or ECZ spectrometer. Chemical shifts and couplings constants of strongly coupled spin systems are given as simulated by *gNMR*.[3] Crystal data was collected with Mo*K*α radiation on a Bruker D8 Venture diffractometer with a CMOS area detector. Single crystals were picked at −40 °C under nitrogen atmosphere and mounted on a 0.15 mm Micromount using perfluoroether oil. The structure was solved with the ShelXT[4] structure solution program using intrinsic phasing and refined with the ShelXL[5] refinement package using least squares on weighted F2 values for all reflections using OLEX2.[6] CCDC 2154970 and CCDC 2153771 contain the supplementary crystallographic data for this paper. These data are provided free of charge by The Cambridge Crystallographic Data Centre. Cyclic voltammetry was performed on an Interface 1010 B Potentiostat/Galvanostat/ZRA from Gamry Instruments. The investigations were carried out starting from 0 V going to the reduction first and then to the oxidation. The measurements were performed at a scan rate of 100 mV/s in anhydrous solvents under argon atmosphere without extra supporting and platinum wires as working-, counter-, and quasi-reference electrodes. The voltammograms were internally referenced against $Fc^{0/+}$. The compound was freshly prepared in the cyclic voltammetry cell with a concentration of 0.078 M in *ortho*-difluorobenzene. The Turbomole program[7] was used to perform calculations at the unrestricted Kohn-Sham DFT level, using the B3LYP hybrid functional[8] (with RI[9]) in conjunction with the valence triple-ζ basis set with two sets of polarization functions (def2-TZVPP)[10]. Minima on potential energy surfaces were characterized by normal mode analysis. Thermochemical data is provided without counterpoise correction but including zero-point energy correction as obtained from harmonic vibrational frequencies.

$C(C_6F_5)_3OH$

In a Schlenk flask fine magnesium powder (2.43 g, 100 mmol, 3 eq.) was suspended in diethylether (150 ml) and cooled to 0 °C. Bromopentafluorobenzene (24.7, 100 mmol, 3 eq.) was added dropwise and the mixture was allowed to warm to room temperature. After 4 hours, methyl chloroformate (3.15 g, 33 mmol, 1 eq.) was added in small portions and the reaction mixture was consecutively stirred for 36 hours at room temperature, finally followed by 4 hours stirring under reflux conditions. Afterwards, the mixture was treated with diluted HCl solution (10 %, 20 ml) and then extracted with diethyl ether (3x 30 ml). The collected organic phases were washed with dist. water (3x 30 ml) and Brine solution (3x 30 ml). After drying with $MgSO_4$, all volatiles were removed under reduced pressure. The resulting crude oil was then refined via fractionated sublimation. The first fraction (50 °C, $1*10^{-3}$ mbar) belongs to the sideproduct decafluorobenzophenone. The desired product was collected at 100 °C and $1*10^{-3}$ mbar as yellow crystals (6.73 g, 35 %). ^{1}H NMR (400 MHz, $CDCl_3$, 22 °C): *δ* = 4.29 (s, 1H, -O*H*) ppm; ^{19}F NMR (377 MHz, $CDCl_3$, 22 °C): *δ* = –140.0 (m, 6 *ortho*-F), –151.0 (m, 3 *para*-F), –160.2 (m, 6 *meta*-F) ppm.

These data are in agreement with literature values of $C(C_6F_5)_3OH$.[11]

$C(C_6F_5)_3Cl$

This synthesis is a modified version of an already reported procedure.[11]

Tris(pentafluorophenyl)methanol $C(C_6F_5)_3OH$ (3.53 g, 7 mmol) was dissolved in thionyl chloride (25.00 g) resulting in a yellow solution. Pyridine (0.49 g, 7 mmol, 1 eq.) and dimethylformamide (0.52 g, 7 mmol, 1 eq.) were added and the mixture was brought to reflux at 80 °C for 48 hours under constant stirring. Afterwards, the mixture was cooled to room temperature and decanted on ice water. It was treated with small portions of saturated $NaHCO_3$ solution until the formation of gas stopped. The aqueous phase was extracted with dichloromethane (3x 30 ml). The collected organic phases were subsequently washed with saturated $NaHCO_3$ and Brine solution (each 3x 30 ml), dried with $MgSO_4$ and finally all volatiles were removed under reduced pressure. After recrystallization in *n*-pentane at –16 °C the product was obtained as yellow powder (0.95 g, 22 %). ^{19}F NMR (377 MHz, $CDCl_3$, 22 °C): δ = –135.5 (m, 6 *ortho*-F), –150.0 (m, 3 *para*-F), –160.3 (m, 6 *meta*-F) ppm.

These data are in agreement with literature values of $C(C_6F_5)_3Cl$.[11]

$[C(C_6F_5)_3][Al(OTeF_5)_4]$

Triethylaluminum (41.7 mg, 0.37 mmol, 1 eq.) was dissolved in 4 ml of *ortho*-difluorobenzene and degassed. Afterwards, pentafluoroorthotelluric acid $HOTeF_5$ (350 mg, 1.46 mmol, 4 eq.) was condensed onto the frozen solution at –196 °C. Warming the mixture to –30 °C under constant stirring led to a clear yellow solution and gas evolution. After 10 minutes the gas evolution ceased and tris(pentafluorophenyl)methyl chloride (200 mg, 0.37 mmol, 1 eq.) was added via a funnel. The solution turned intense purple and a further gas evolution could be observed. Stock solutions can be prepared and directly used. NMR spectra were recorded at –40 °C. ^{19}F NMR (282 MHz, $C_6H_4F_2$, ext. [D$_6$]acetone, –40 °C): δ = –40.1 (m, $1F_A$, $^2J(^{19}F_A,^{19}F_B)$=186 Hz, $^1J(^{19}F_A,^{125}Te)$=3158 Hz), –47.2 (m, $4F_B$, $^1J(^{19}F_B,^{125}Te)$=3471 Hz), –112.6 (m, 3 *para*-F), –127.7 (m, 6 *ortho*-F), –154.3 (m, 6 *meta*-F) ppm. ^{27}Al NMR (78 MHz, $C_6H_4F_2$, ext. [D$_6$]acetone, –40 °C): δ = 47 (s, $[Al(OTeF_5)_4]^-$) ppm.

$[C(C_6F_5)_3][Al(OTeF_5)_3Cl]$

In a J. Young NMR tube $[Al(OTeF_5)_3]_2$ (38 mg, 0.0025 mmol, 0.5 eq) was weighed in and SO_2ClF was condensed on top at −196 °C. Warming to −30 °C led to a clear, colorless solution. Afterwards, tris(pentafluoro-phenyl)methyl chloride (28 mg, 0.05 mmol) was added. Shaking of the mixture led to an intense purple-colored solution, which was analyzed by low-temperature NMR spectroscopy. ^{19}F NMR (377 MHz, SO_2ClF, ext. [D$_6$]acetone, –60 °C): δ = –38.9 (m, $1F_A$, $^2J(^{19}F_A,^{19}F_B)$=176 Hz), –40.0 (m, $1F_{A'}$, $^2J(^{19}F_{A'},^{19}F_{B'})$=182 Hz), –46.1 (m, $4F_B$, $^1J(^{19}F_B,^{125}Te)$=3467 Hz), –46.5 (m, $4F_{B'}$, $^1J(^{19}F_{B'},^{125}Te)$=3463 Hz) –112.6 (m, 3 *para*-F), –127.7 (m, 6 *ortho*-F), –154.3 (m, 6 *meta*-F) ppm; ^{27}Al NMR (78 MHz, SO_2ClF, ext. [D$_6$]acetone, –60 °C): δ = 80 (s, $[Al(OTeF_5)_2Cl_2]^-$), 63 (s, $[Al(OTeF_5)_3Cl]^-$), 48 (s, $[Al(OTeF_5)_4]^-$) ppm; ^{13}C NMR (101 MHz, SO_2ClF, ext. [D$_6$]acetone, –60 °C): 175.3 (central C), 155.6 (*para*-C), 149.0 (*ortho*-C), 140.2 (*meta*-C), 115.6 (*ipso*-C) ppm.

Reaction of $[C(C_6F_5)_3][Al(OTeF_5)_4]$ with isobutane

The reaction was performed in a J. Young NMR tube. To a cooled solution of $[Al(OTeF_5)_3]_2$ (44 mg, 0.03 mmol, 0.5 eq.) in SO_2ClF at −60 °C, tris(pentafluorophenyl)methyl chloride (33 mg, 0.06 mmol, 1 eq.) was added under an argon stream, forming a deep purple solution. Finally, isobutane (7 mg, 0.12 mmol, 4 eq.) was condensed on the mixture at −196 °C. The reaction mixture was then brought to –60 °C and further warming was monitored and analyzed with low-temperature NMR spectroscopy

Reaction of $[C(C_6F_5)_3][Al(OTeF_5)_4]$ with ferrocene

A stock-solution of $[C(C_6F_5)_3][Al(OTeF_5)_4]$ in *ortho*-difluorobenzene (0.05 mmol/ml) at –30 °C was treated with solid ferrocene (9 mg, 0.05 mmol). Upon contact the solution immediately changed its color to blue and further reacted to a green solution, indicating the successful formation of the ferrocenium cation. The mixture was analyzed by EPR spectroscopy

Reaction of $[C(C_6F_5)_3][Al(OTeF_5)_4]$ with tris-(4-bromophenyl)amine

A stock-solution of $[C(C_6F_5)_3][Al(OTeF_5)_4]$ in *ortho*-difluorobenzene (0.052 mmol/ml) at –30 °C was treated with solid tris-(4-bromophenyl)amine (13 mg, 0.052 mmoll). Upon contact the solution immediately changed its color to a dark blue, indicating the formation of the radical cationic ammoniumyl. The mixture was analyzed by EPR spectroscopy.

NMR Spectra

$C(C_6F_5)_3OH$

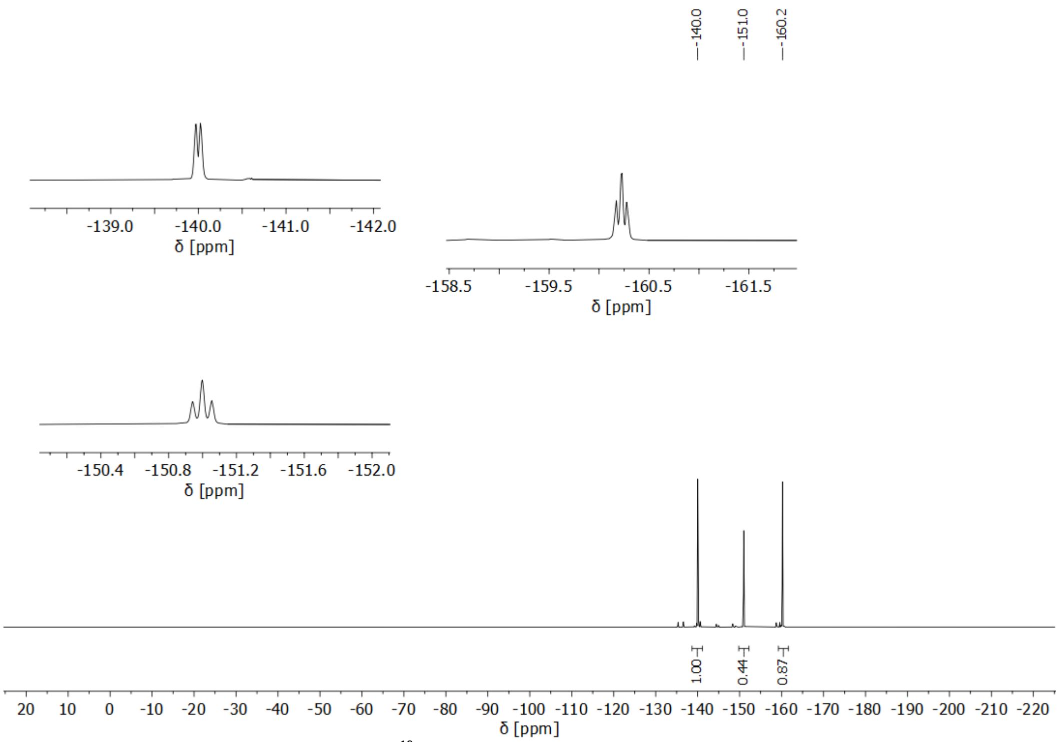

Figure ^{19}F **S1**. NMR (377 MHz, $CDCl_3$, 22 °C).

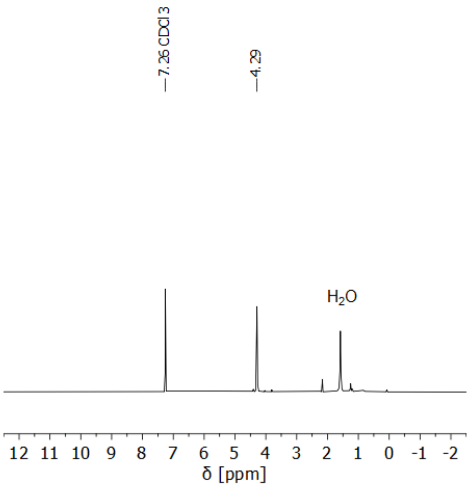

Figure S2. ^1H NMR (400 MHz, $CDCl_3$, 22 °C).

$C(C_6F_5)_3Cl$

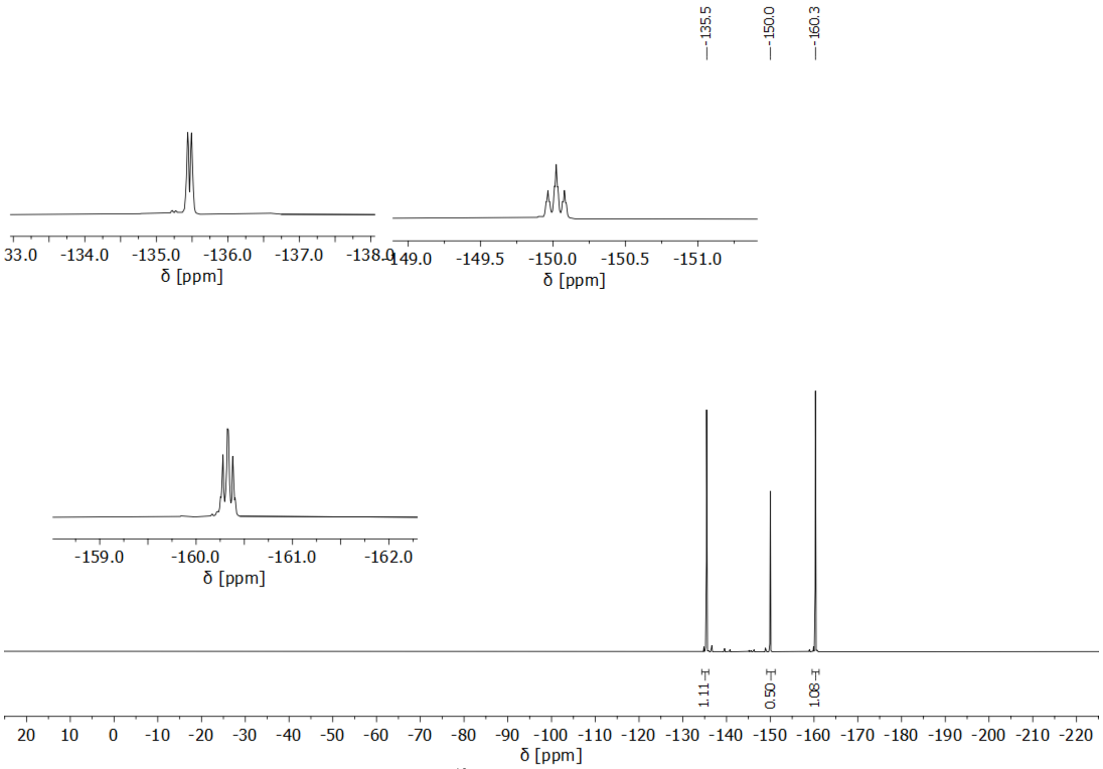

Figure S3. ^{19}F NMR (377 MHz, $CDCl_3$, 22 °C).

[C(C6F5)3][Al(OTeF5)4]

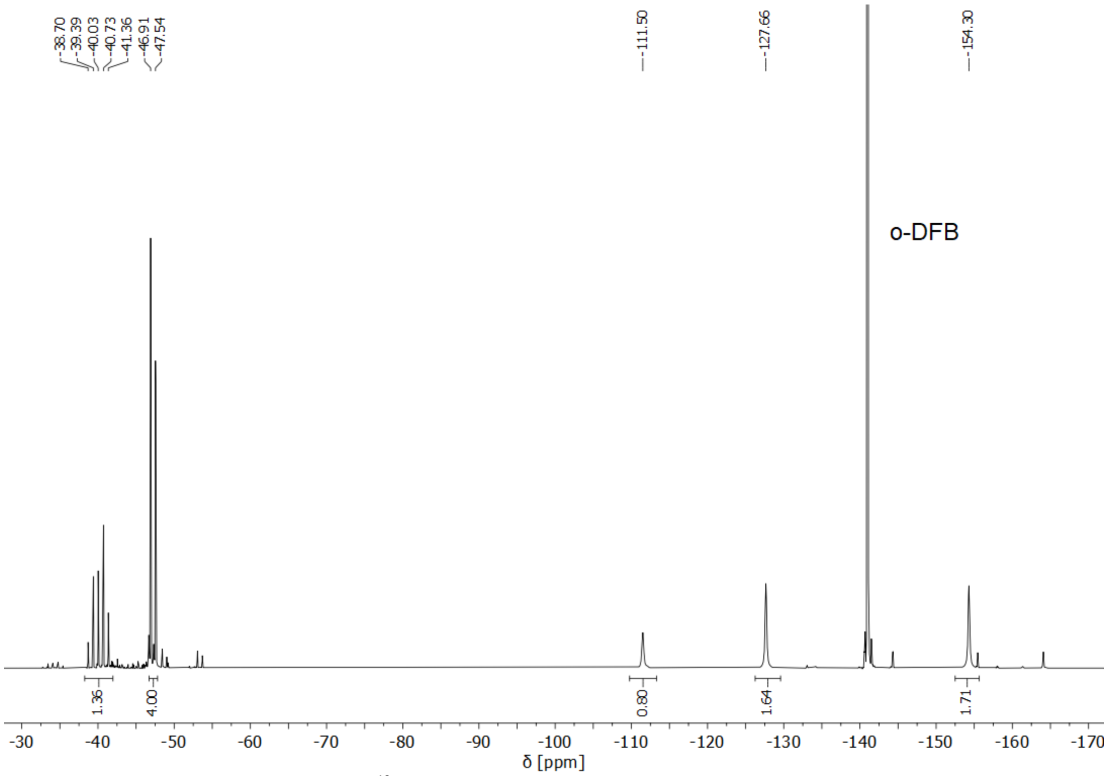

Figure S4. ^{19}F NMR (377 MHz, $C_6H_4F_2$, ext. [D6]acetone, –40 °C).

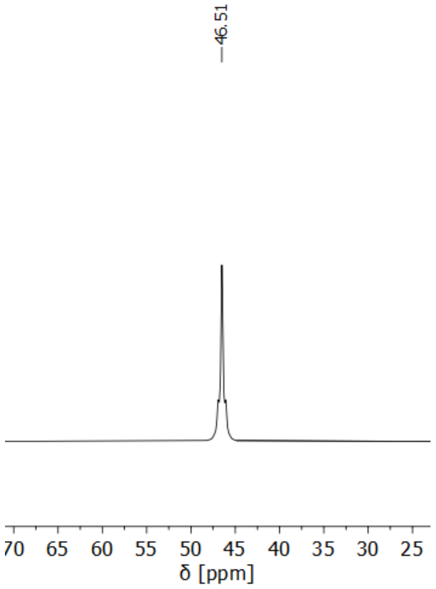

Figure S5. ^{27}Al NMR (78 MHz, $C_6H_4F_2$, ext. [D6]acetone, –40 °C).

[C(C6F5)3][Al(OTeF5)3Cl]

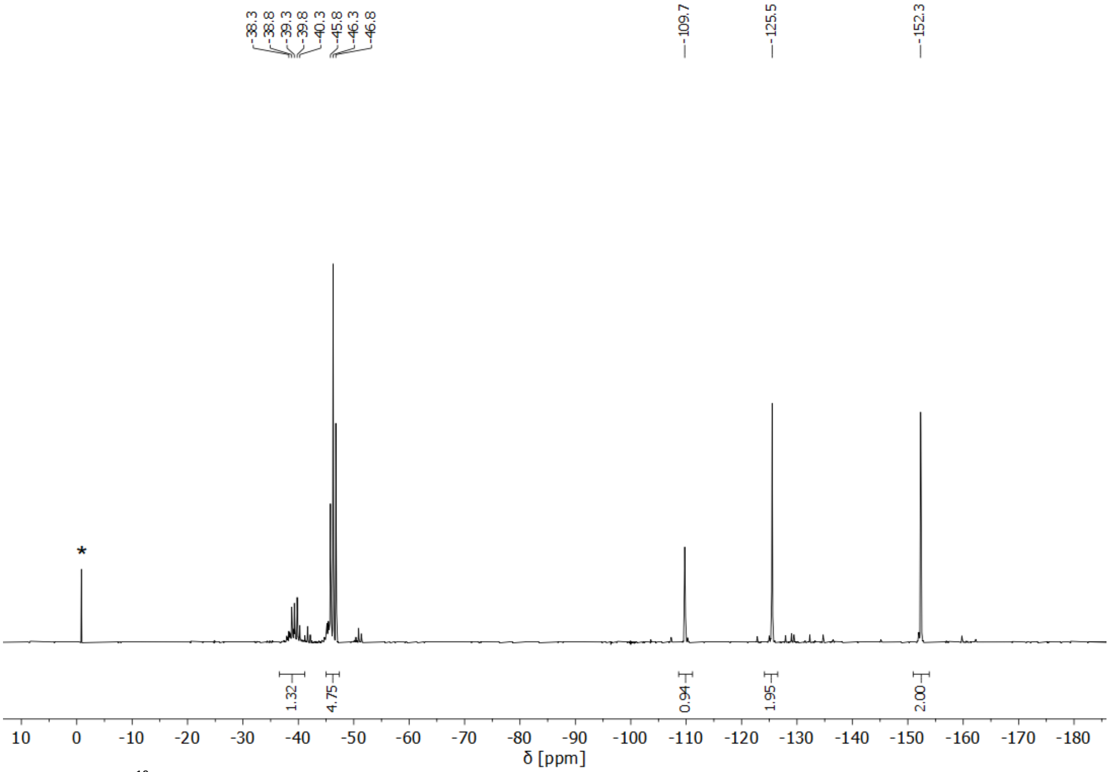

Figure S6. ^{19}F NMR (377 MHz, SO_2ClF, ext. [D6]acetone, –60 °C). The asterisk marks the signal of the [D6]acetone capillary.

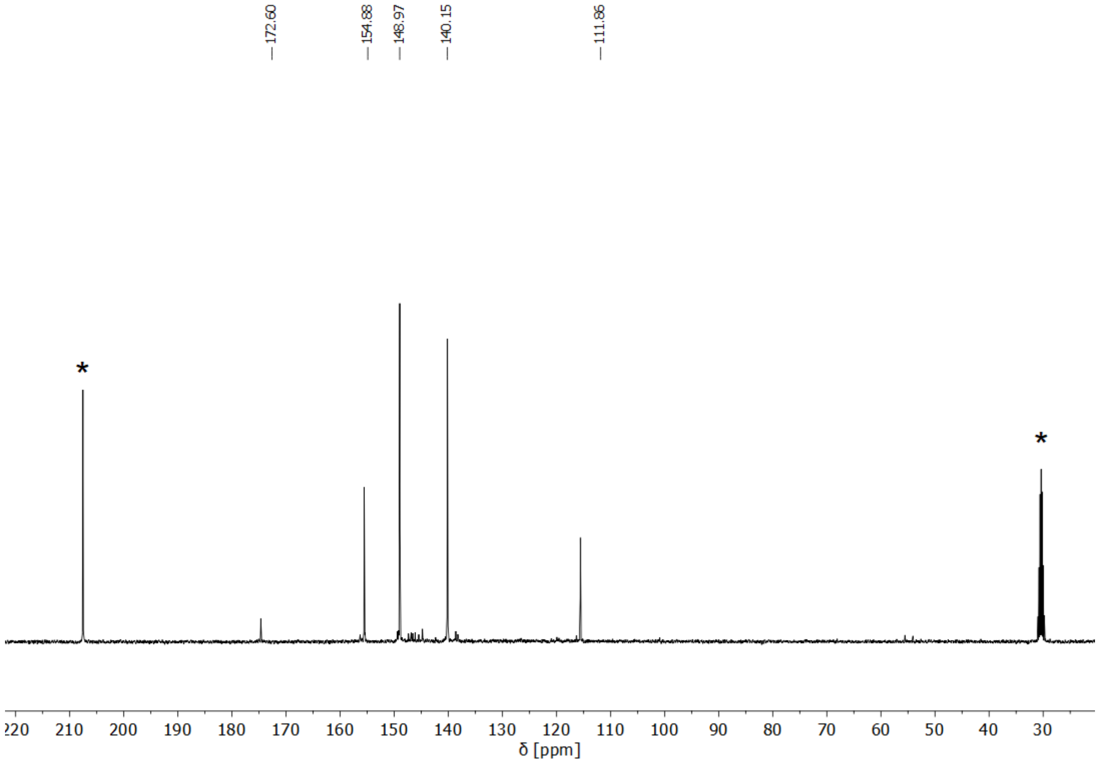

Figure S7. ^{13}C NMR (101 MHz, SO_2ClF, ext. [D6]acetone, –60 °C). Asterisks mark the signals of the [D6]acetone capillary.

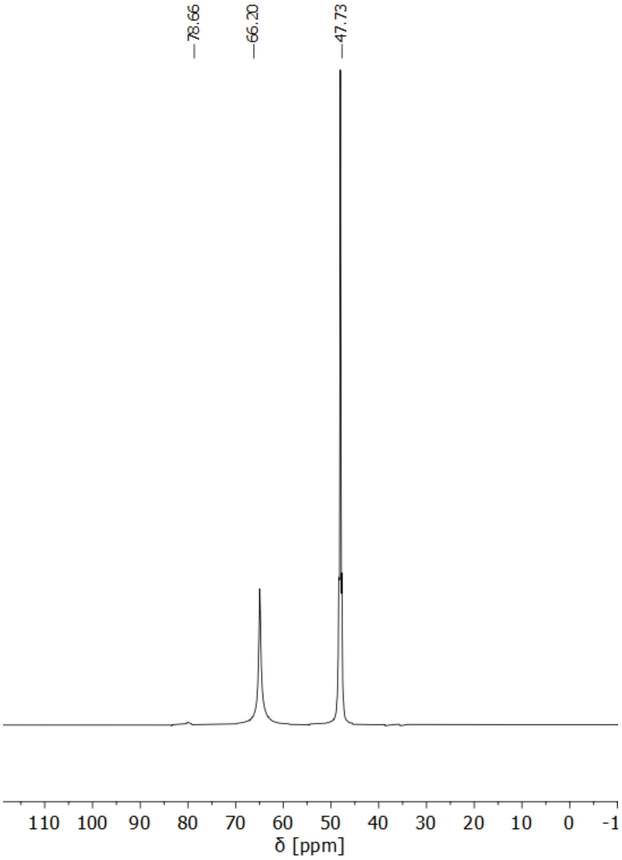

Figure S8. ^{27}Al NMR (78 MHz, SO_2ClF, ext. [D_6]acetone, –60 °C).

Decomposition of [C(C_6F_5)$_3$][Al($OTeF_5$)$_4$] in *o*-DFB at room temperature

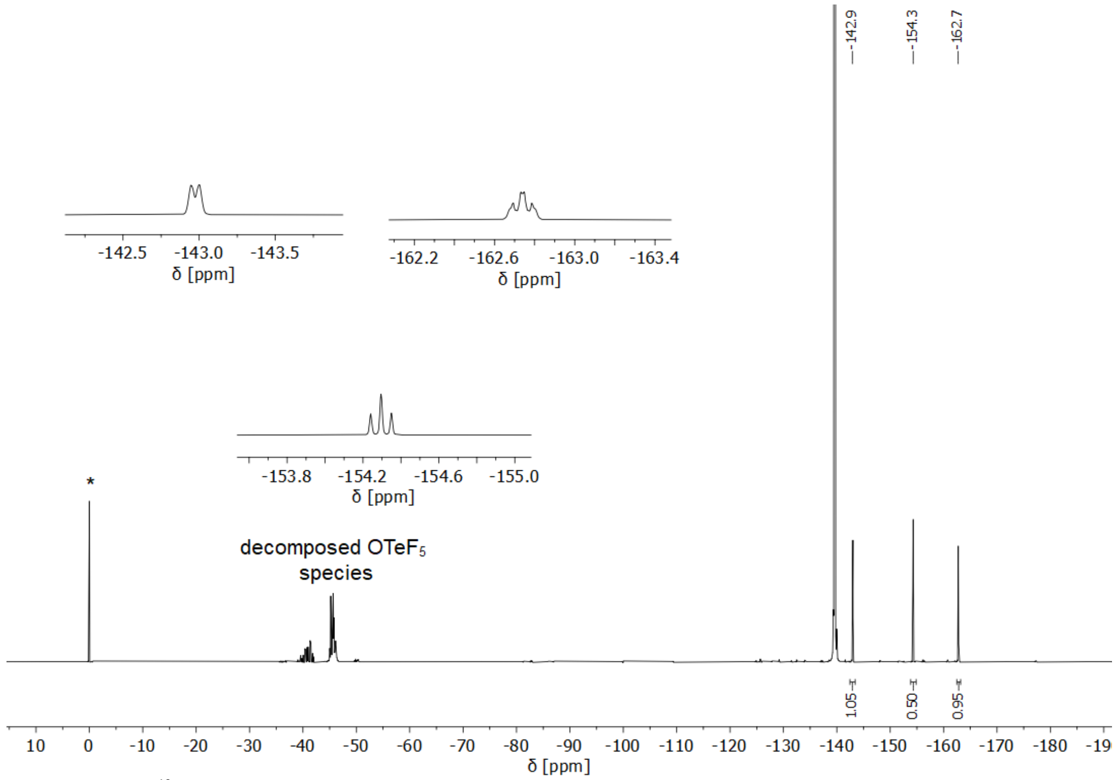

Figure S9. ^{19}F NMR (377 MHz, $C_6H_4F_2$, ext. [D_6]acetone, 22 °C). Asterisks marks the signals of the [D_6]acetone capillary

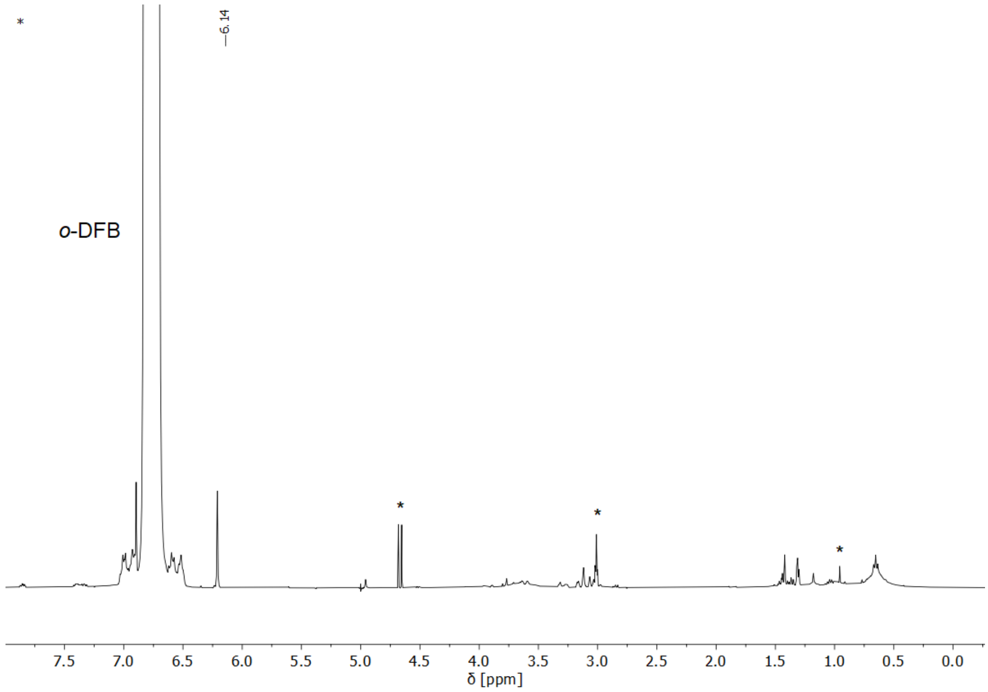

Figure S10. ^{19}F NMR (377 MHz, $C_6H_4F_2$, ext. [D_6]acetone, 22 °C). Asterisks mark the signals of the [D_6]acetone capillary.

Variable-Temperature NMR Spectra

After the sample has been warmed for several hours to 0 °C, the sample was measured again at –60 °C. The ^{19}F NMR spectrum reveals the complete conversion of cation $[C(C_6F_5)_3]^+$ to perfluorotrityl methane. Furthermore, the formation of $HOTeF_5$ was observed in the ^{19}F and ^1H NMR spectra, probably occurring due to partial decomposition of $[Al(OTeF_5)_{4-x}Cl_x]^-$ via the protonation of the Brønsted acidic *tert*-butyl cation. Similar decomposition reactions have been reported by Krossing et al. when they treated $AlBr_3$ with *tert*-butylbromide.[12] The broadened signals of the isobutane in the ^1H and ^{13}C NMR spectra are resolved again, suggesting that the dynamic exchange ceased. Additionally, several signals with low intensity in the range of 1 to 3 ppm in the ^1H NMR and 0 to 50 ppm in the ^{13}C NMR are observed, which correspond to isobutene oligomerization products. Still, no clearly assignable signals of the *tert*-butyl cation are found, which most likely reacted with the excess isobutane of the reaction mixture. Additional experiments were performed in which a slight excess of $HOTeF_5$ is added to the reaction of the Lewis acid $Al(OTeF_5)_3(SO_2ClF)$ and $C(C_6F_5)_3Cl$ in SO_2ClF in order to form HCl and therefore remove the chloride source before the isobutane is added. Furthermore, an equimolar amount of isobutane was used. These attempts also resulted in the rapid oligomerization of the formed *tert*-butyl cation at 0 °C and finally yielded the polymerization of the sample at room temperature.

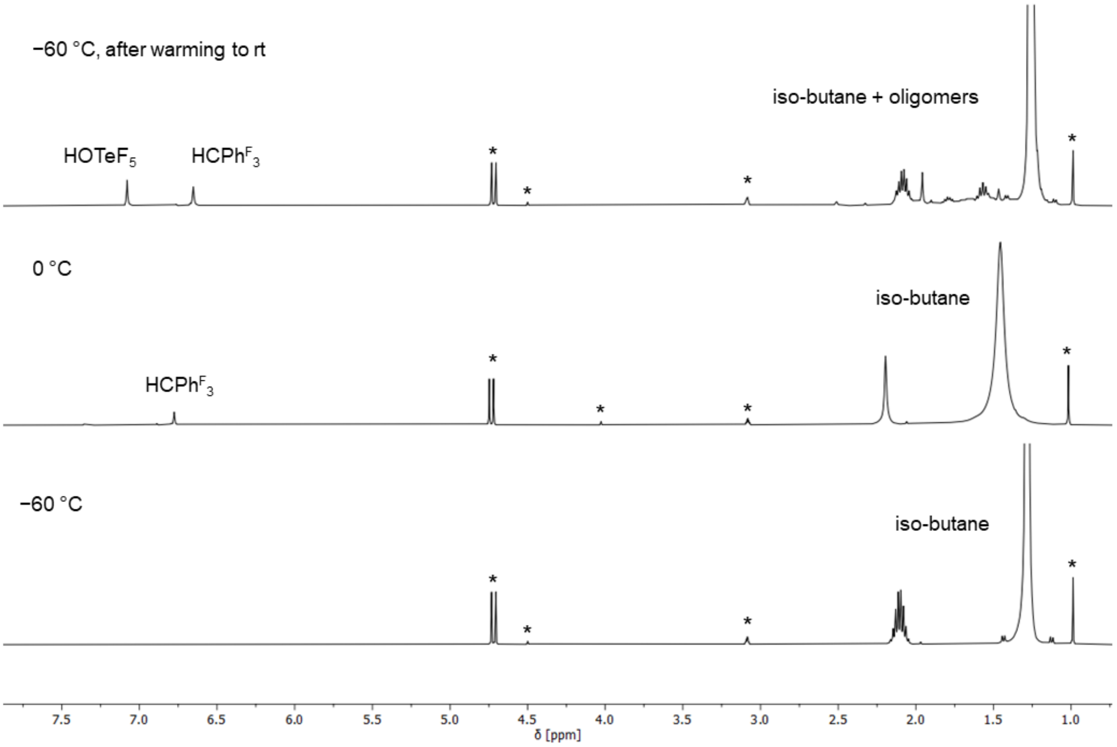

Figure S11. VT ^1H NMR (400 MHz, SO_2ClF, ext. [D6]acetone) spectra of the reaction of $[CPh^F{}_3][Al(OTeF_5)_3Cl]$ with isobutane. Asterisk marks the signal of the external deuterated solvent capillary.

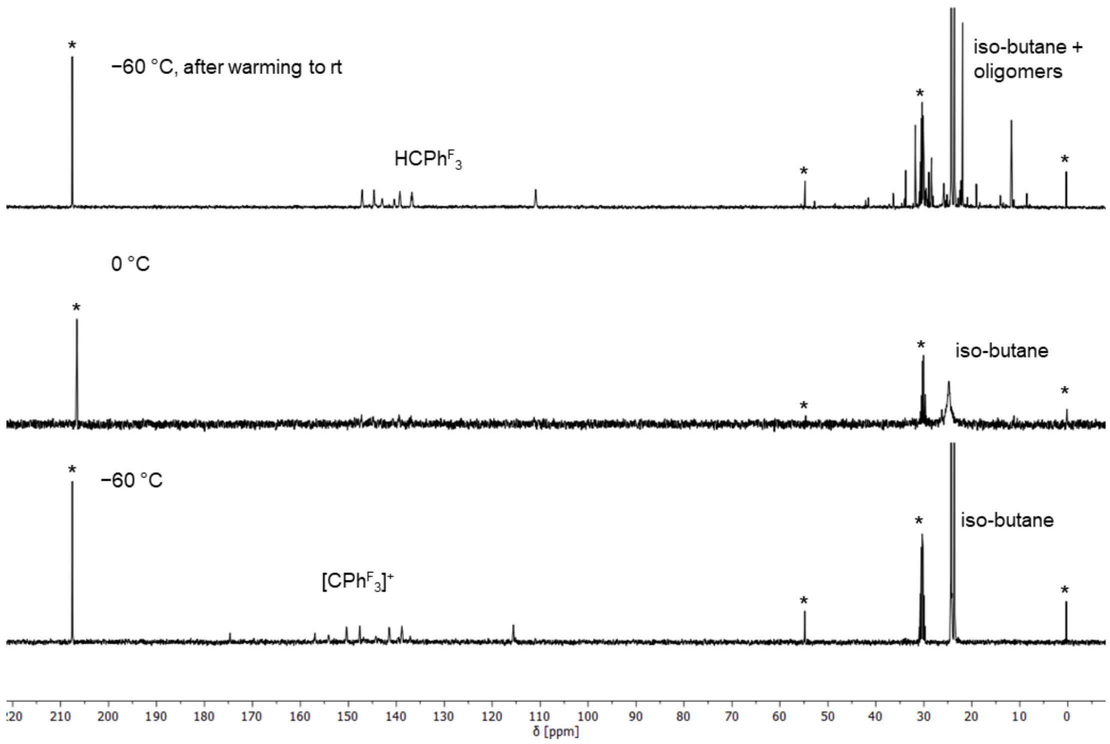

Figure S12. VT ^{13}C NMR (101 MHz, SO_2ClF, ext. [D$_6$]acetone) spectra of the reaction of [CPh^F_3][$Al(OTeF_5)_3Cl$] with isobutane. Asterisk marks the signal of the external deuterated solvent capillary.

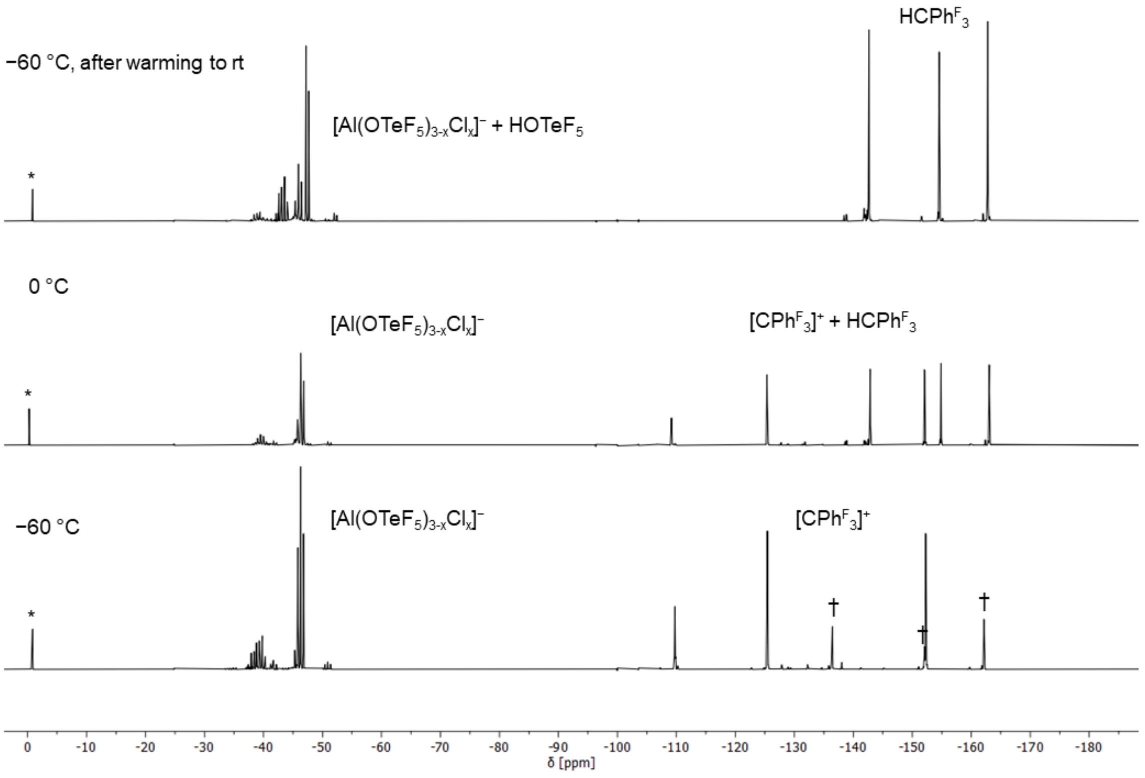

Figure S13. VT ^{19}F NMR (282 MHz, SO_2ClF, ext. [D$_6$]acetone) spectra of the reaction of [CPh^F_3][$Al(OTeF_5)_3Cl$] with isobutane. Asterisk denotes the signal of the external deuterated solvent capillary. Daggers denotes residual CPh^F_3Cl.

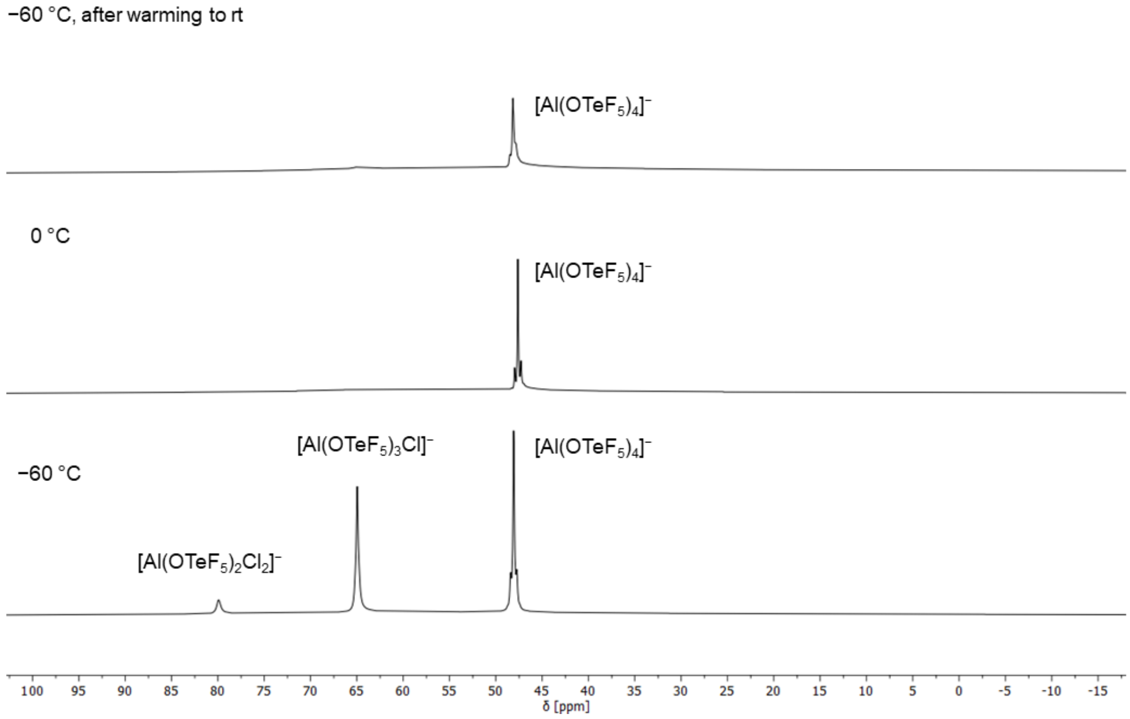

Figure S14. VT ^{27}Al NMR (78 MHz, SO_2ClF, ext. [D$_6$]acetone) spectra of the reaction of [CPh^F_3][$Al(OTeF_5)_3Cl$] with isobutane. Asterisk marks the signal of the external deuterated solvent capillary.

EPR Spectra

$$[Ph^{F}{}_3C][Al(OTeF_5)_4] + FeCp_2 \xrightarrow[-30\ °C \rightarrow r.t.]{SET,\ o\text{-}DFB} Ph^{F}{}_3C^{\bullet} + [FeCp_2][Al(OTeF_5)_4]$$

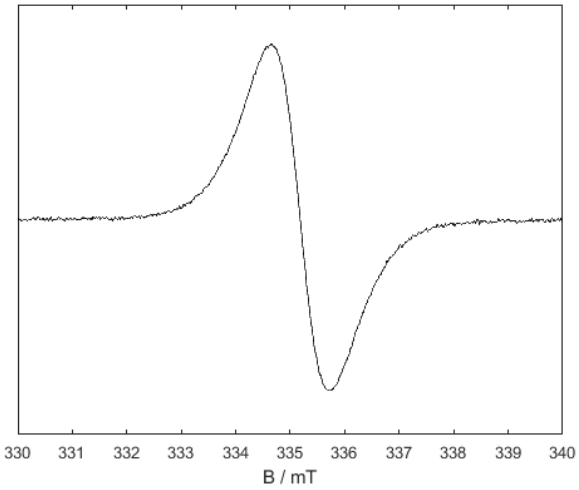

Figure S15. Experimental EPR spectrum showing the signal of the perfluorinated trityl radical $CPh^{F}{}_3$ at room temperature. The spectrum was recorded using the solution obtained after the reaction of $[CPh^{F}{}_3][Al(OTeF_5)_4]$ and ferrocene. The experimental *g* value is 2.0031.

$$[Ph^{F}{}_3C][Al(OTeF_5)_4] + N(C_6H_4Br\text{-}4)_3 \xrightarrow[-30\ °C \rightarrow r.t.]{SET,\ o\text{-}DFB} Ph^{F}{}_3C^{\bullet} + [N(C_6H_4Br\text{-}4)_3][Al(OTeF_5)_4]$$

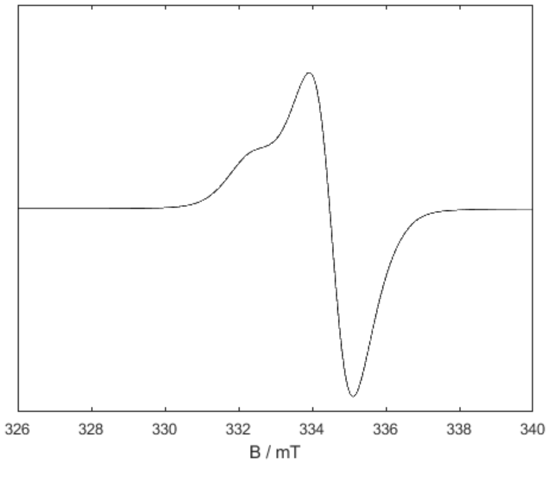

Figure S16. Experimental EPR spectrum showing two broad overlapped signals of the perfluorinated trityl radical $CPh^{F}{}_3$ and the ammoniumyl radical cation $[N(C_6H_4Br\text{-}4)_3][Al(OTeF_5)_4]$ at room temperature. The spectrum was recorded using the solution obtained after the reaction of $[CPh^{F}{}_3][Al(OTeF_5)_4]$ and the amine $N(C_6H_4Br\text{-}4)_3$.

Crystal data

Summary of crystal data and refinement results

	$C(C_6F_5)_3H$	$[C(C_6F_5)_3][Al(OTeF_5)_4]$
CCDC number	2154970	2153771
empirical formula	$C_{19}HF_{15}$	$AlC_{19}F_{35}O_4Te_4$
formula weight	514.20	1494.57
temperature [K]	100	100
crystal system	trigonal	monoclinic
space group	$R\bar{3}$	*C2/c*
a [pm]	3785.7(4)	1383.99(18)
b [pm]	3785.7(4)	2029.23(18)
c [pm]	610.10(7)	1400.13(15)
α [°]	90	90
β [°]	90	115.648(5)
γ [°]	120	90
volume [Å^3]	7572.2(17)	3544.7(7)
Z	18	4
ρ_{calcd} [g · cm^{-3}]	2.030	2.801
μ [mm^{-1}]	0.236	3.500
dimension [mm]	0.949 × 0.058 × 0.052	0.459 × 0.164 × 0.03
color	colorless	colorless
reflection collected	20405	105750
independent reflections	3096 [R_{int} = 0.0692]	3642 [R_{int} = 0.0467]
data/retrains/parameters	3096/0/307	3642/612/363
goodness-of-fit on F^2	0.993	1.147
final *R* indexes [$I > 2\sigma(I)$]	R_1 = 0.0463<br>wR_2 = 0.1069	R_1 = 0.0349<br>wR_2 = 0.0763
final *R* indexes [all data]	R_1 = 0.0734<br>wR_2 = 0.1216	R_1 = 0.0388<br>wR_2 = 0.0786

$C(C_6F_5)_3H$

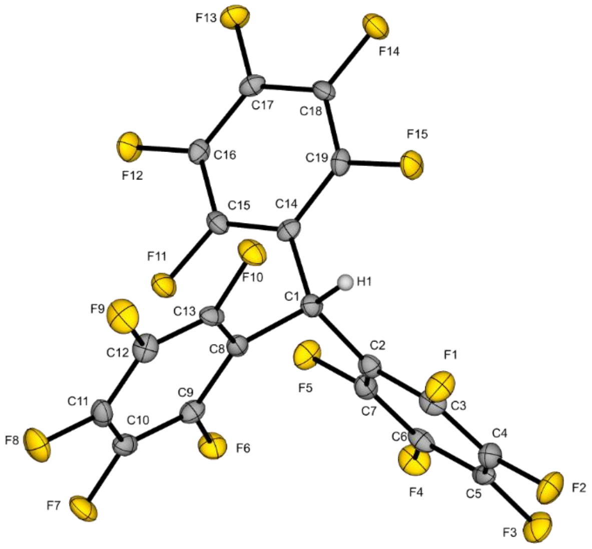

Figure S17. Molecular structure of $C(C_6F_5)_3H$.

Table S1. Bond Lengths for $C(C_6F_5)_3H$.

Atom	Atom	Length/Å	Atom	Atom	Length/Å
F1	C3	1.346(3)	C2	C3	1.382(4)
F2	C4	1.335(3)	C2	C7	1.387(4)
F3	C5	1.336(3)	C3	C4	1.381(4)
F4	C6	1.347(3)	C4	C5	1.377(4)
F5	C7	1.344(3)	C5	C6	1.380(4)
F6	C9	1.343(3)	C6	C7	1.375(4)
F7	C10	1.343(3)	C8	C9	1.388(4)
F8	C11	1.342(3)	C8	C13	1.385(4)
F9	C12	1.338(3)	C9	C10	1.386(4)
F10	C13	1.342(3)	C10	C11	1.370(4)
F11	C15	1.342(3)	C11	C12	1.379(4)
F12	C16	1.345(3)	C12	C13	1.382(4)
F13	C17	1.341(3)	C14	C15	1.383(4)
F14	C18	1.337(3)	C14	C19	1.384(4)
F15	C19	1.350(3)	C15	C16	1.381(4)
C1	C2	1.535(4)	C16	C17	1.374(4)
C1	C8	1.527(4)	C17	C18	1.377(4)
C1	C14	1.536(4)	C18	C19	1.372(4)

Table S2. Bond Angles for $C(C_6F_5)_3H$.

Atom	Atom	Atom	Angle/˚	Atom	Atom	Atom	Angle/˚
C2	C1	C14	113.5(2)	C11	C10	C9	119.9(3)
C8	C1	C2	114.6(2)	F8	C11	C10	120.3(3)
C8	C1	C14	113.4(2)	F8	C11	C12	120.1(3)
C3	C2	C1	118.7(3)	C10	C11	C12	119.6(3)
C3	C2	C7	116.0(3)	F9	C12	C11	120.0(3)
C7	C2	C1	125.4(3)	F9	C12	C13	120.6(3)
F1	C3	C2	119.8(3)	C11	C12	C13	119.4(3)
F1	C3	C4	117.0(3)	F10	C13	C8	119.9(3)
C4	C3	C2	123.2(3)	F10	C13	C12	117.0(3)

F2	C4	C3	120.7(3)	C12	C13	C8	123.1(3)
F2	C4	C5	120.1(3)	C15	C14	C1	125.3(3)
C5	C4	C3	119.2(3)	C15	C14	C19	115.7(3)
F3	C5	C4	120.6(3)	C19	C14	C1	119.0(3)
F3	C5	C6	120.2(3)	F11	C15	C14	120.7(3)
C4	C5	C6	119.3(3)	F11	C15	C16	117.0(3)
F4	C6	C5	119.8(3)	C16	C15	C14	122.3(3)
F4	C6	C7	120.0(3)	F12	C16	C15	120.4(3)
C7	C6	C5	120.2(3)	F12	C16	C17	119.8(3)
F5	C7	C2	120.4(3)	C17	C16	C15	119.8(3)
F5	C7	C6	117.4(3)	F13	C17	C16	119.7(3)
C6	C7	C2	122.2(3)	F13	C17	C18	120.5(3)
C9	C8	C1	125.5(3)	C16	C17	C18	119.8(3)
C13	C8	C1	118.9(3)	F14	C18	C17	119.9(3)
C13	C8	C9	115.6(3)	F14	C18	C19	121.1(3)
F6	C9	C8	120.4(3)	C19	C18	C17	119.0(3)
F6	C9	C10	117.1(3)	F15	C19	C14	119.4(3)
C10	C9	C8	122.5(3)	F15	C19	C18	117.1(3)
F7	C10	C9	120.0(3)	C18	C19	C14	123.4(3)
F7	C10	C11	120.1(3)				

Table S3. Hydrogen Atom Coordinates (Å×10[4]) and Isotropic Displacement Parameters (Å[2]×10[3]) for $C(C_6F_5)_3H$.

Atom	x	y	z	U(eq)
H1	6442.03	5169.25	7875.25	18

[C(C$_6$F$_5$)$_3$][Al(OTeF$_5$)$_4$]

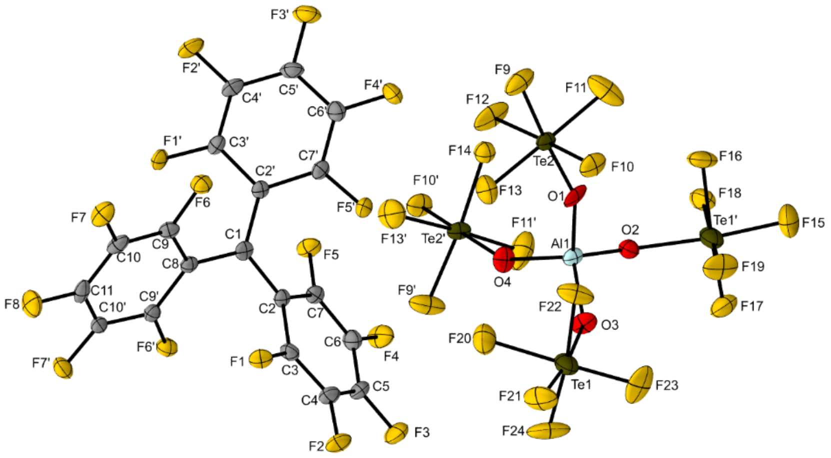

Figure S18. Molecular structure of [C(C$_6$F$_5$)$_3$][Al(OTeF$_5$)$_4$].

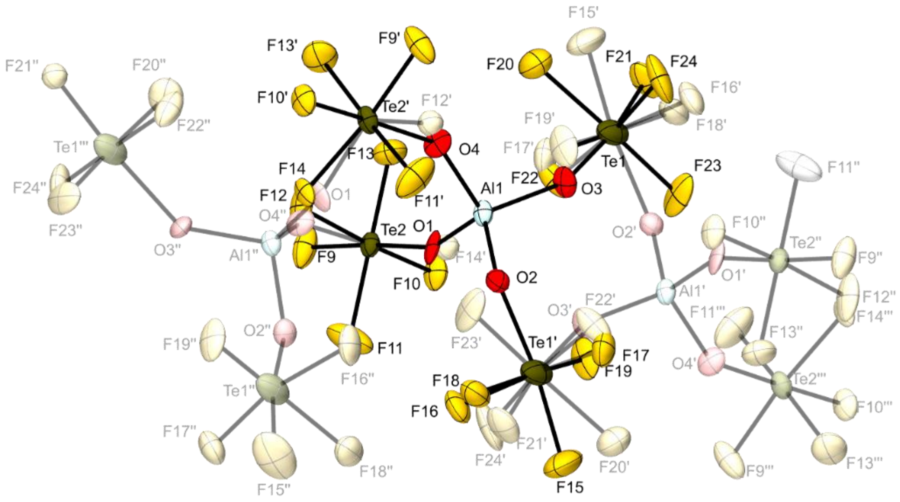

Figure S19. Molecular structure of the disordered anion in [C(C$_6$F$_5$)$_3$][Al(OTeF$_5$)$_4$].

Table S4. Bond Lengths for [C(C$_6$F$_5$)$_3$][Al(OTeF$_5$)$_4$].

Atom	Atom	Length/Å	Atom	Atom	Length/Å
Te2	F10	1.835(3)	F7	C10	1.333(6)
Te2	F9	1.839(3)	F5	C7	1.325(6)
Te2	F13	1.820(4)	F2	C4	1.334(6)
Te2	O1	1.78(3)	F4	C6	1.323(6)
Te2	F11	1.820(4)	F8	C11	1.319(9)
Te2	O4^1	1.88(2)	F3	C5	1.319(6)
Te2	F12	1.774(19)	Al1	O1	1.68(3)
Te2	F14	1.85(3)	Al1	O4	1.718(17)
Te1	F22	1.814(17)	Al1	O3	1.713(10)
Te1	O3^2	1.985(9)	Al1	O2	1.731(10)

Te1	F16	1.870(8)	C8	C9[3]	1.416(6)
Te1	O2	1.864(9)	C8	C9	1.416(6)
Te1	F18	2.008(9)	C8	C1	1.449(10)
Te1	F20	1.938(8)	C9	C10	1.371(7)
Te1	F15	1.789(9)	C2	C1	1.432(6)
Te1	F23	1.703(9)	C2	C3	1.430(7)
Te1	F19	1.604(9)	C2	C7	1.413(7)
Te1	F21	1.719(8)	C10	C11	1.390(7)
Te1	F24	1.825(9)	C4	C5	1.381(8)
Te1	F17	1.825(18)	C4	C3	1.371(7)
F6	C9	1.332(6)	C6	C5	1.386(8)
F1	C3	1.318(6)	C6	C7	1.378(7)

[1]1-X,+Y,3/2-Z; [2]1/2-X,3/2-Y,1-Z; [3]-X,+Y,1/2-Z

Table S5. Bond Angles for $[C(C_6F_5)_3][Al(OTeF_5)_4]$.

Atom	Atom	Atom	Angle/˚	Atom	Atom	Atom	Angle/˚
F10	Te2	F9	85.37(15)	F21	Te1	F22	90.7(7)
F10	Te2	O4[1]	175.6(7)	F21	Te1	O3[2]	172.1(5)
F10	Te2	F14	85.1(7)	F21	Te1	F20	87.0(5)
F9	Te2	O4[1]	93.8(9)	F21	Te1	F24	90.9(4)
F9	Te2	F14	170.5(7)	F24	Te1	O3[2]	87.3(5)
F13	Te2	F10	88.52(17)	F24	Te1	F20	86.7(5)
F13	Te2	F9	88.85(19)	F17	Te1	O3[2]	96.0(8)
F13	Te2	F11	174.99(19)	F17	Te1	F16	167.0(8)
F13	Te2	O4[1]	95.8(6)	F17	Te1	O2	90.4(5)
F13	Te2	F14	91.1(13)	F17	Te1	F18	83.7(8)
O1	Te2	F10	94.4(9)	O1	Al1	O4	107.9(18)
O1	Te2	F9	176.7(17)	O1	Al1	O3	114.9(11)
O1	Te2	F13	94.4(17)	O1	Al1	O2	107.2(18)
O1	Te2	F11	89.4(17)	O4	Al1	O2	110.8(8)
F11	Te2	F10	87.96(18)	O3	Al1	O4	108.0(11)
F11	Te2	F9	87.3(2)	O3	Al1	O2	108.0(5)
F11	Te2	O4[1]	87.7(6)	Al1	O1	Te2	156(3)
F11	Te2	F14	92.1(13)	C9	C8	C9[3]	115.9(6)
F12	Te2	F10	170.5(6)	C9	C8	C1	122.1(3)
F12	Te2	F9	87.3(8)	C9[3]	C8	C1	122.1(3)
F12	Te2	F13	85.2(6)	F6	C9	C8	120.1(5)
F12	Te2	F11	97.8(7)	F6	C9	C10	117.7(5)
F12	Te2	O4[1]	12.3(10)	C10	C9	C8	122.1(5)
F12	Te2	F14	102.2(11)	C3	C2	C1	121.7(5)
F14	Te2	O4[1]	95.7(11)	C7	C2	C1	122.3(5)
F22	Te1	O3[2]	90.2(7)	C7	C2	C3	116.0(4)
F22	Te1	F20	86.2(6)	F7	C10	C9	120.6(5)
F22	Te1	F24	172.6(7)	F7	C10	C11	119.4(5)
O3[2]	Te1	F18	162.8(4)	C9	C10	C11	120.0(5)
F16	Te1	O3[2]	97.0(4)	C2[3]	C1	C8	119.8(3)
F16	Te1	F18	83.8(4)	C2	C1	C8	119.8(3)
O2	Te1	O3[2]	77.8(4)	C2	C1	C2[3]	120.5(6)
O2	Te1	F16	92.0(4)	F8	C11	C10	120.1(3)
O2	Te1	F18	84.9(4)	F8	C11	C10[3]	120.1(3)
F20	Te1	O3[2]	85.3(4)	C10	C11	C10[3]	119.7(7)
F15	Te1	O3[2]	114.7(5)	F2	C4	C5	119.9(5)
F15	Te1	F16	87.6(4)	F2	C4	C3	119.7(5)
F15	Te1	O2	167.4(5)	C3	C4	C5	120.4(5)
F15	Te1	F18	82.6(5)	F4	C6	C5	119.5(5)
F15	Te1	F17	87.2(5)	F4	C6	C7	121.0(5)
F23	Te1	F22	93.7(6)	C7	C6	C5	119.6(5)

F23	Te1	O3[2]	92.3(4)	F3	C5	C4	119.8(5)
F23	Te1	F20	177.6(4)	F3	C5	C6	119.8(5)
F23	Te1	F21	95.5(5)	C4	C5	C6	120.4(5)
F23	Te1	F24	93.4(6)	F1	C3	C2	119.8(4)
F19	Te1	F22	96.3(8)	F1	C3	C4	118.8(5)
F19	Te1	O3[2]	19.0(5)	C4	C3	C2	121.3(5)
F19	Te1	F20	67.9(5)	F5	C7	C2	120.3(4)
F19	Te1	F23	109.8(5)	F5	C7	C6	117.5(5)
F19	Te1	F21	153.2(5)	C6	C7	C2	122.2(5)
F19	Te1	F24	79.1(5)	Al1	O2	Te1	133.9(6)

[1]1-X,+Y,3/2-Z; [2]1/2-X,3/2-Y,1-Z; [3]-X,+Y,1/2-Z

Table S6. Atomic Occupancy for $[C(C_6F_5)_3][Al(OTeF_5)_4]$.

Atom	Occupancy	Atom	Occupancy	Atom	Occupancy
Al1	0.5	O1	0.5	O4	0.5
F22	0.5	O3	0.5	F16	0.5
O2	0.5	F18	0.5	F20	0.5
F15	0.5	F23	0.5	F19	0.5
F21	0.5	F24	0.5	F17	0.5
F12	0.5	F14	0.5		

Quantum-chemical calculations

Structure optimizations were performed DFT level using the B3LYP functional together with def2-TZVPP basis set as implemented in the Turbomole V7.3 program.

CPh_3 radical

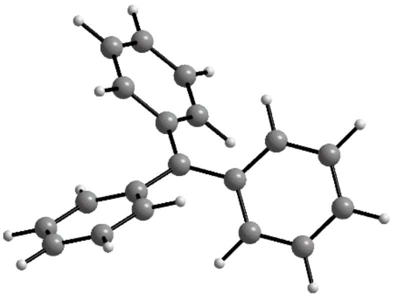

Figure S20. Representation of the B3LYP/def2-TZVPP structure of the CPh_3 radical.

```
$coord
   -3.13196863839529     -0.02141756615702      1.71721899799721  c
   -4.95758580017693     -2.02464255822518      2.23663245826561  c
   -7.58005563073335     -1.60577260294866      2.01265258089143  c
   -8.24785073380689      0.22825474950869      1.40953456077638  h
   -9.31053342158524     -3.51161881920265      2.49520960704563  c
  -11.30997974031826     -3.13724087759807      2.27732750314509  h
   -8.49234458559724     -5.89968458029861      3.23856910898188  c
   -9.84576075519705     -7.38364367144206      3.62202133278227  h
   -5.91234860498653     -6.35609994453278      3.48292257133340  c
   -5.25134162867099     -8.19774482484253      4.07987157020826  h
   -4.17528334390754     -4.46065945430963      2.98305653779683  c
   -2.18084585620237     -4.84008554594961      3.21039918270288  h
    1.97979776656735     -4.65570604630747     -3.69722113727487  h
    1.86123266209212     -3.16120310151079     -2.30523145472153  c
   -0.45151812048952     -2.59187384561587     -1.21567487947800  c
   -2.11269786619500     -3.63863338557835     -1.78008446243604  h
   -0.69532589410538     -0.63369240214670      0.57557353599355  c
    1.51525355351575      0.70993196989295      1.21604325954300  c
    1.40585375765980      2.20059738375591      2.60877193458144  h
    3.82849266366415      0.12148116789596      0.13782134391145  c
    5.49549985376024      1.16978344438648      0.69214102075028  h
    4.01991607613048     -1.81404894212709     -1.63457060291808  c
    5.82539359798795     -2.26645930664567     -2.48091030050482  h
   -1.82133105506026      4.21401008418346     -0.86641276628079  h
   -2.90719258346174      4.61838839824585      0.81622732221251  c
   -3.49616069281751      7.10201912483922      1.40406675552794  c
   -2.85049964962966      8.60864766516790      0.18000905229480  h
   -4.92218729753315      7.66398295894094      3.54304922565541  c
   -5.37341581683585      9.60411962662592      4.00466205182288  h
   -5.76149074098822      5.70066950506029      5.08121766269939  c
   -6.85340299153553      6.11285031919587      6.76133307797971  h
   -5.19442134343275      3.21340534922376      4.48752188300241  c
   -5.83770509116921      1.71463856703204      5.71776770897705  h
   -3.74371161607381      2.59771147620367      2.33876698657065  c
$end
```

E_{tot}= −732.8281357854 H

[CPh3]+ cation

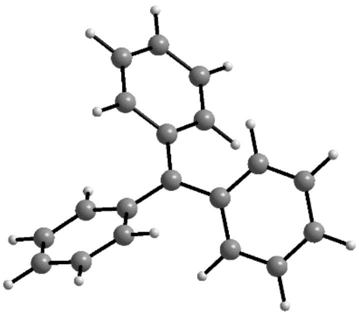

Figure S21. Representation of the B3LYP/def2-TZVPP structure of the [CPh3]+ cation.

```
$coord
   -3.13034876138922    -0.01950510799536     1.71921643722896  c
   -4.93667097029271    -2.00217547292532     2.23427640107620  c
   -7.56142615766595    -1.54944506711499     2.01312763734952  c
   -8.21826237519897     0.27715563159430     1.38132241869735  h
   -9.28100992260958    -3.46130662601394     2.46939856155231  c
  -11.27994157298732    -3.11054280103302     2.23328211175193  h
   -8.43716478978566    -5.84185041511979     3.22013344183803  c
   -9.79023975749378    -7.32656850586361     3.60031668544937  h
   -5.85949591666082    -6.32011104379028     3.48377102706351  c
   -5.21673419586087    -8.15844246734918     4.10135232144907  h
   -4.12208401869730    -4.43992550273455     2.96729501192002  c
   -2.12925208365253    -4.80054210010024     3.22399918212504  h
    1.92765959179923    -4.64949337237787    -3.68665614167781  h
    1.79735236620714    -3.16546307729286    -2.28847083796031  c
   -0.50477824529713    -2.61531297825473    -1.18520928618285  c
   -2.17471002389962    -3.64123059359770    -1.75623776751548  h
   -0.72080910939945    -0.62681974034324     0.58790023611610  c
    1.47681428310243     0.75473591184393     1.22512023176900  c
    1.36609777017015     2.22788590635518     2.63390908436641  h
    3.78225074223647     0.14795412497247     0.15904186664863  c
    5.45963162459463     1.17832116976508     0.70580979958482  h
    3.94521270004884    -1.79872447958049    -1.60918503955378  c
    5.74865460182843    -2.25161662109356    -2.45892035451986  h
   -1.79184670887454     4.16916313501642    -0.88389243869892  h
   -2.85914241392603     4.58147718142127     0.80673811408130  c
   -3.46926681287654     7.05360027306703     1.38715499401252  c
   -2.84015182199898     8.57005673736874     0.17107854881186  h
   -4.90474587143507     7.58845829454833     3.53110560978148  c
   -5.35577086895620     9.52792740028145     3.99442593152401  h
   -5.76277854666247     5.63805622716645     5.08130308691422  c
   -6.84537593373683     6.06526602754480     6.76044768365275  h
   -5.21913799275807     3.15551756953903     4.47997803218178  c
   -5.84186622162223     1.65245820931114     5.71289630674012  h
   -3.73618215377697     2.57130648750529     2.33445433225770  c
$end
```

E_{tot}= −732.6127398496 H

CPh$^{F}_{3}$ radical

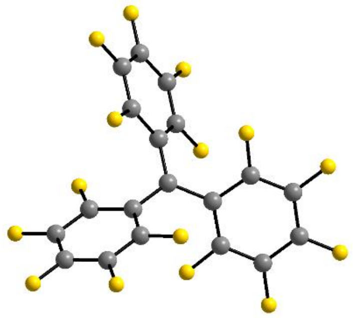

Figure 22. Representation of the B3LYP/def2-TZVPP structure of the CPh$^{F}_{3}$ radical.

```
$coord
    0.00102095831423    -0.00589087565973     0.00801778726910  c
   -1.82152910352303    -2.00315931051489     0.52915276113432  c
   -4.43323105393526    -1.67697743004412     0.15706131329673  c
   -5.31850549875523     0.50218192407784    -0.77007250157835  f
   -6.17810287468856    -3.56283025959276     0.63717200121991  c
   -8.62878580200458    -3.16711531176416     0.21575467539219  f
   -5.36753163106176    -5.89476886542917     1.52911723906414  c
   -7.02895482441862    -7.71831447986014     1.99088592776722  f
   -2.80578995638073    -6.30272472566063     1.93179279976384  c
   -2.01525359585412    -8.52118038427255     2.82184047457524  f
   -1.09170826741715    -4.39216659209148     1.43746138651539  c
    1.34096786557728    -4.86998780281962     1.93380340273554  f
    5.11338473085629    -4.61814072727159    -6.08996900924995  f
    4.98429096674587    -2.96340067833226    -4.19634556549664  c
    2.68226141947498    -2.37693480373256    -3.10387667783727  c
    0.62791561438334    -3.50556932012894    -4.05296779517895  f
    2.43839720887598    -0.61749352355453    -1.12979196677633  c
    4.69917423469118     0.51753472758734    -0.32120866889103  c
    4.67250507230553     2.15924744734229     1.60218728524416  f
    7.02142446834353    -0.04699214089245    -1.38208137458523  c
    9.11831689998071     1.04879006848443    -0.52000482766640  f
    7.17165126120826    -1.79471394485263    -3.33492310989912  c
    9.39103715683998    -2.34469567169809    -4.37072032839057  f
    1.12063294546648     4.16350061591777    -3.22901776693544  f
    0.00368782745405     4.62472560410400    -1.00768626785207  c
   -0.58009162635164     7.11325441834269    -0.45708087298921  c
   -0.00190468529490     8.94866705430707    -2.08153229558881  f
   -1.81008875951849     7.69728580483153     1.78691195431761  c
   -2.37705057227108    10.08017695996412     2.33389844398078  f
   -2.44325268381658     5.77127430161393     3.45474743394851  c
   -3.59416579889494     6.32311275995127     5.62526990152057  f
   -1.84697705325195     3.29541895740893     2.86476601460146  c
   -2.43771390546845     1.52338037113236     4.56959801605902  f
   -0.60603093761067     2.61450583310680     0.61784021050972  c
$end
```

E_{tot}= −2221.462296850 H

$[CPh^{F}{}_3]^+$ cation

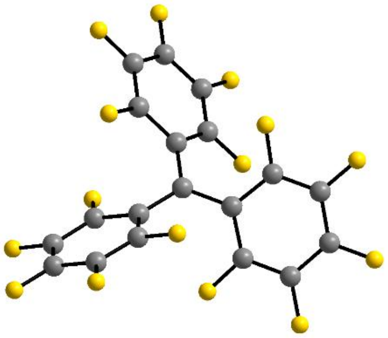

Figure S23. Representation of the B3LYP/def2-TZVPP structure of the $[CPh^{F}{}_3]^+$ cation.

```
$coord
   -3.12847519778563     -0.03427606137065      1.72822146445654  c
   -4.93970743187294     -2.00629125346953      2.24176378330425  c
   -7.57167506998977     -1.63373020801220      1.89791216029448  c
   -8.43311735217169      0.54493905178775      1.00250632940774  f
   -9.31262289323390     -3.52285039764603      2.33214446182964  c
  -11.74229083640152     -3.13778722756079      1.91425185350927  f
   -8.48889728953204     -5.86887697891823      3.21654009059876  c
  -10.13442534025011     -7.66061877466915      3.66030297845973  f
   -5.91947402989705     -6.31256399319108      3.62790083103769  c
   -5.16620700503527     -8.52319616055764      4.50571671300607  f
   -4.19512200180034     -4.42885981279714      3.11171431447322  c
   -1.77922089307287     -4.91241597336640      3.58083012896927  f
    1.98668030845786     -4.79764930398260     -4.19919776577930  f
    1.82389897630195     -3.07487925705258     -2.40167098406411  c
   -0.46237958531801     -2.53657382455660     -1.27258836654440  c
   -2.47986441698428     -3.77538471489228     -2.10188177407108  f
   -0.71930991579581     -0.64041815020000      0.60525100344119  c
    1.52978858760846      0.64239357502560      1.30113032051966  c
    1.48682920641729      2.36991152179240      3.11993133194211  f
    3.84080214759853      0.07745818737314      0.23885093761327  c
    5.90840592234744      1.26364083598089      0.97611253926368  f
    3.98916447665892     -1.77394782350118     -1.63631641566980  c
    6.16923684157296     -2.29337699265672     -2.68141490042123  f
   -1.82839198058644      4.14333222829216     -1.41859522728765  f
   -3.00786570801213      4.59123590385105      0.75020519429517  c
   -3.60165136180392      7.06665543564855      1.30627131551841  c
   -2.96416723176018      8.91305743657836     -0.24660955709049  f
   -4.89774339521536      7.61984890794082      3.53780073314571  c
   -5.43621440399136      9.96804261456755      4.09599795765257  f
   -5.62412508625126      5.68206545665473      5.17574327924069  c
   -6.81549428911881      6.23353655532936      7.29767368322188  f
   -5.08209391360525      3.20976241926809      4.55584553040179  c
   -5.75685065859233      1.43203069927408      6.19281422377032  f
   -3.73291984915130      2.55605039375651      2.33512506139000  c
$end
```

E_{tot}= −2221.192766570 H

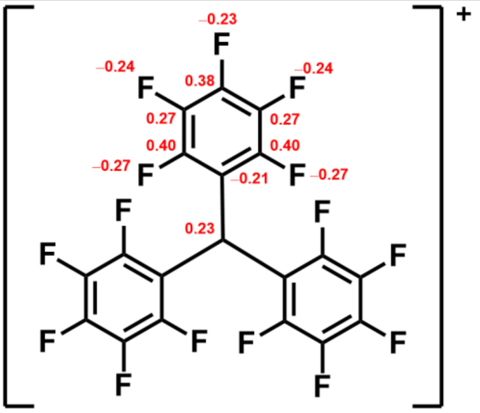

Figure S24. Visualization of the NPA charges

atomic populations from total density:

atom	charge	n(s)	n(p)	n(d)	n(f)	n(g)
1 c	0.22815	2.90596	2.85875	0.00548	0.00166	0
2 c	-0.207	2.92786	3.2709	0.00557	0.00267	0
3 c	0.39863	2.86748	2.72383	0.00829	0.00177	0
4 f	0.26632	3.82418	0.43424	0.00777	0.00013	0
5 c	0.26857	2.8775	2.84235	0.00967	0.00191	0
6 f	0.24373	3.82387	0.41166	0.00806	0.00014	0
7 c	0.37481	2.89338	2.72071	0.0094	0.00169	0
8 f	0.22447	3.82019	0.3953	0.00884	0.00015	0
9 c	0.26906	2.87763	2.84175	0.00966	0.00191	0
10 f	-0.24368	3.82381	5.41167	0.00807	0.00014	0
11 c	0.39892	2.86712	2.72388	0.0083	0.00177	0
12 f	-0.26632	3.82416	5.43426	0.00777	0.00013	0
13 f	-0.24371	3.82381	5.41169	0.00807	0.00014	0
14 c	0.26878	2.8777	2.84193	0.00968	0.00191	0
15 c	0.39917	2.86763	2.72315	0.00828	0.00177	0
16 f	-0.26642	3.82426	5.43427	0.00776	0.00013	0
17 c	-0.20669	2.92816	3.27029	0.00558	0.00267	0
18 c	0.3985	2.86756	2.72386	0.00831	0.00177	0
19 f	-0.26662	3.82429	5.43444	0.00776	0.00013	0
20 c	0.2691	2.87757	2.84176	0.00966	0.00191	0
21 f	-0.24366	3.82383	5.41163	0.00806	0.00014	0
22 c	0.37503	2.89337	2.72047	0.00943	0.00169	0
23 f	-0.2244	3.82016	5.39526	0.00884	0.00015	0
24 f	-0.26712	3.82431	5.43493	0.00775	0.00013	0
25 c	0.39747	2.86721	2.72524	0.0083	0.00177	0
26 c	0.26926	2.87758	2.84159	0.00966	0.00191	0
27 f	-0.24385	3.82388	5.41178	0.00806	0.00014	0
28 c	0.37376	2.89323	2.7219	0.00942	0.0017	0
29 f	-0.22485	3.82017	5.3957	0.00883	0.00015	0
30 c	0.26909	2.87756	2.84177	0.00967	0.00191	0
31 f	-0.24389	3.82383	5.41186	0.00806	0.00014	0
32 c	0.39793	2.86734	2.72467	0.00829	0.00177	0
33 f	-0.26677	3.82436	5.43453	0.00775	0.00013	0
34 c	-0.20672	2.9279	3.27056	0.00559	0.00267	0

$HCPh^F_3$

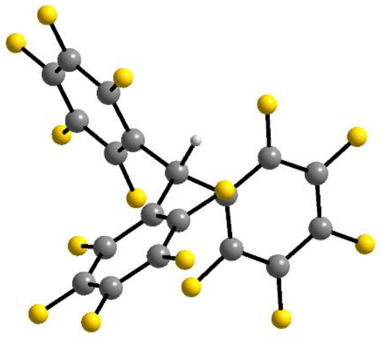

Figure S25. Representation of the B3LYP/def2-TZVPP structure of $HCPh^F_3$.

```
$coord
  -0.43242750805016     0.11287100728542    -1.01694465034275  c
  -2.03922444607518    -1.97147747845669     0.16868531523283  c
  -4.56318751821306    -2.16573178580534    -0.56172538675342  c
  -5.50559024607474    -0.51515944116360    -2.23850245870965  f
  -6.17522204512185    -4.00559037141251     0.37377033186685  c
  -8.57426308385832    -4.11283501738654    -0.38362839475288  f
  -5.26455474365669    -5.74718980062378     2.10934125747594  c
  -6.78299659498517    -7.52329601938330     3.03271351842612  f
  -2.76161228374391    -5.61741055609950     2.87728256756725  c
  -1.87524775217661    -7.27387751117889     4.55376308290948  f
  -1.19361834355298    -3.74700264944628     1.91460353496209  c
   1.18577059940600    -3.68975019177612     2.76416569249142  f
   5.66679864136353    -3.79340607972783    -6.68647479870586  f
   5.25788775266411    -2.58676493590162    -4.51436962413502  c
   2.83042230996776    -1.90288992240869    -3.80530357976236  c
   0.93091392594808    -2.50220258913728    -5.37314293914444  f
   2.30422714258549    -0.62917780341081    -1.55964520559647  c
   4.36772797027345    -0.07802330144498    -0.02385752962301  c
   4.04938722075826     1.09262165442053     2.19291663899325  f
   6.82036336628722    -0.72980133396256    -0.68916647605809  c
   8.74261365993196    -0.16368854162681     0.83610426678460  f
   7.27029121808692    -1.99039727979459    -2.94262556619541  c
   9.61319589769076    -2.62677455878355    -3.58879923853098  f
  -0.44128468939166     4.57468105473029    -3.81470785010133  f
  -0.75698432718067     4.83741817862691    -1.31363995693545  c
  -1.08992883108848     7.26050465379448    -0.37522951178299  c
  -1.09160322572448     9.24686308057988    -1.92259140054532  f
  -1.43196528911954     7.60345149216663     2.20177901000285  c
  -1.75348334528894     9.91196736078589     3.13928524184582  f
  -1.42966355507435     5.52178526101219     3.79431517485305  c
  -1.74182486865912     5.83801019698880     6.27295761217810  f
  -1.08222775561832     3.11850065993386     2.80180878998869  c
  -1.05702930550392     1.19090881276050     4.43581567537661  f
  -0.74367695006078     2.69981515017280     0.22705915311520  c
  -1.25198299674452     0.36304860567308    -2.88601229639481  h
$end
```

E_{tot}= −-2222.093075202 H

References

[1] K. Seppelt, D. Nothe, *Inorg. Chem.* **1973**, *12*, 2727.
[2] A. Wiesner, T. W. Gries, S. Steinhauer, H. Beckers, S. Riedel, *Angew. Chem. Int. Ed. Engl.* **2017**, *56*, 8263; *Angew. Chem.* **2017**, *129*, 8375.
[3] Adept Scientific, *gNMR V 5.0*, **2005**.
[4] G. M. Sheldrick, *Acta Crystallogr. A* **2008**, *64*, 112.
[5] G. M. Sheldrick, *Acta Cryst. (Acta Crystallographica)* **2015**, *C71*, 3.
[6] O. V. Dolomanov, L. J. Bourhis, R. J. Gildea, J. A. K. Howard, H. Puschmann, *J. Appl. Cryst.* **2009**, *42*, 339.
[7] TURBOMOLE GmbH, *TURBOMOLE V7.3. a development of University of Karlsruhe and Forschungszentrum Karlsruhe*, **2018**.
[8] a) A. D. Becke, *Phys. Rev. A* **1988**, *38*, 3098; b) C. Lee, W. Yang, R. G. Parr, *Phys. Rev. B* **1988**, *37*, 785; c) S. H. Vosko, L. Wilk, M. Nusair, *Can. J. Phys.* **1980**, *58*, 1200.
[9] M. Sierka, A. Hogekamp, R. Ahlrichs, *J. Chem. Phys.* **2003**, *118*, 9136.
[10] F. Weigend, R. Ahlrichs, *Phys. Chem. Chem. Phys.* **2005**, *7*, 3297.
[11] E. G. Delany, S. Kaur, S. Cummings, K. Basse, D. J. D. Wilson, J. L. Dutton, *Chem. Eur. J.* **2019**, *25*, 5298.
[12] F. Scholz, D. Himmel, H. Scherer, I. Krossing, *Chem. Eur. J.* **2013**, *19*, 109.
